# Immunoinformatics-aided rational design of multiepitope-based peptide vaccine (MEBV) targeting human parainfluenza virus 3 (HPIV-3) stable proteins

**DOI:** 10.1186/s43141-023-00623-5

**Published:** 2023-12-06

**Authors:** Md Sakib Hossen, Md. Nazmul Hasan, Munima Haque, Tawsif Al Arian, Sajal Kumar Halder, Md. Jasim Uddin, M. Abdullah-Al-Mamun, Md Salman Shakil

**Affiliations:** 1 Department of Biochemistry and Molecular Biology, Primeasia University, Banani, Dhaka 1213 Bangladesh; 2 Division of Computer Aided Drug Design, BioAid, Mirpur, Dhaka 1216 Bangladesh; 3 Department of Biochemistry and Molecular Biology, Hajee Mohammad Danesh Science and Technology University, Dinajpur, 5200 Bangladesh; 4 Biotechnology Program, Department of Mathematics and Natural Sciences (MNS), Brac University, kha-208, 1 Bir Uttam Rafiqul Islam Ave, Dhaka, 1212 Bangladesh; 5 Department of Pharmacy, Faculty of Biological Sciences, Jahangirnagar University, Savar, Dhaka 1342 Bangladesh; 6 Department of Biochemistry and Molecular Biology, Jahangirnagar University, Savar, Dhaka 1342 Bangladesh; 7 Microbiology Program, Department of Mathematics and Natural Sciences (MNS), Brac University, 66 Mohakhali, Dhaka, 1212 Bangladesh

**Keywords:** Human parainfluenza virus 3, Multiepitope vaccine, Molecular docking, Molecular dynamics simulation, pET32a ( +) vector

## Abstract

**Background:**

Human parainfluenza viruses (HPIVs) are common RNA viruses responsible for respiratory tract infections. Human parainfluenza virus 3 (HPIV-3) is particularly pathogenic, causing severe illnesses with no effective vaccine or therapy available.

**Results:**

The current study employed a systematic immunoinformatic/reverse vaccinology approach to design a multiple epitope-based peptide vaccine against HPIV-3 by analyzing the virus proteome. On the basis of a number of therapeutic features, all three stable and antigenic proteins with greater immunological relevance, namely matrix protein, hemagglutinin neuraminidase, and RNA-directed RNA polymerase L, were chosen for predicting and screening suitable T-cell and B-cell epitopes. All of our desired epitopes exhibited no homology with human proteins, greater population coverage (99.26%), and high conservancy among reported HPIV-3 isolates worldwide. All of the T- and B-cell epitopes are then joined by putative ligands, yielding a 478-amino acid-long final construct. Upon computational refinement, validation, and thorough screening, several programs rated our peptide vaccine as biophysically stable, antigenic, allergenic, and non-toxic in humans. The vaccine protein demonstrated sufficiently stable interaction as well as binding affinity with innate immune receptors TLR3, TLR4, and TLR8. Furthermore, codon optimization and virtual cloning of the vaccine sequence in a pET32a ( +) vector showed that it can be readily expressed in the bacterial system.

**Conclusion:**

The in silico designed HPIV-3 vaccine demonstrated potential in evoking an effective immune response. This study paves the way for further preclinical and clinical evaluation of the vaccine, offering hope for a future solution to combat HPIV-3 infections.

**Supplementary Information:**

The online version contains supplementary material available at 10.1186/s43141-023-00623-5.

## Background

Infections with the human parainfluenza virus (HPIV) are one of the major viral causes of juvenile acute lower respiratory illness (ALRI), which is the second-highest cause of acute respiratory disease–related hospitalizations in children under the age of 5 (1/1000 each year) after influenza [46]. In young children, HPIV-associated ALRI is thought to account for roughly 13% of all ALRI cases worldwide, according to a meta-analysis [83]. In addition to that, 4–14% of hospital admissions with respiratory difficulty along with nearly 4% of childhood mortality were directly related to HPIVs. These figures articulate us how serious HPIV infections can be in young children, causing ALRI morbidities and mortalities [83]. Numerous other investigations have revealed that immunocompromised patients and the elderly are more likely to develop severe ALRI illnesses [11, 52, 56].

Paramyxoviridae members, like HPIVs, are viruses that replicate on a single strand of RNA inside an envelope. Human parainfluenza virus type 3 (HPIV-3) is by far the most often reported strain, followed by types 1 and 2. When HPIV-1 and HPIV-2 are sparse in the springtime and early summertime, it hits its yearly peak and is more frequently accompanied by bronchiolitis and pneumonia [11, 21, 51]. Although legal immunizations to reduce the risk of invasive pneumococcal disease and disease caused by *Haemophilus influenzae* type b are available and are becoming easier to obtain, there is still no registered vaccine available to protect against HPIVs. As a result, an infant HPIV-3 vaccination is necessary and should preferably be administered to newborns as early as 1 month or 2 months of age, because the majority of the concerning illness occurs in the first few months of life. A vaccine that may produce protective immunity in young people is likely to be the most important objective in the development of pediatric respiratory virus vaccines since HPIV-3 can cause an infection very shortly after birth, even in the presence of biologically obtained serum antibodies.

**Fig. 1:**
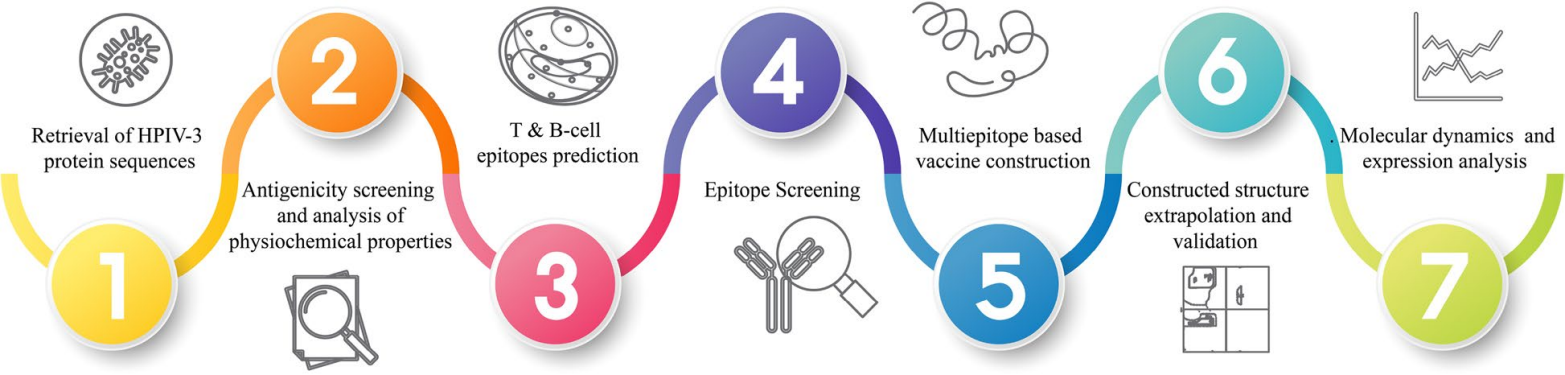
Study protocol for MEBV against HPIV-3 proteome

Vaccination is the gold standard of intervention in the fight against viral infections. Unfortunately, due to a variety of experimental and testing-related shortcomings, traditional approaches to vaccine development are typically extremely difficult, laborious, and costly [54]. In addition, severe allergic, toxic, and autoimmune responses are common with traditionally developed vaccines [10]. Recent advancements in bioinformatics-based platforms provide integrated computational approaches that facilitate fast screening for potential T- and B-cell epitopes, which can be used to develop multiepitope-based vaccines in shorter time with higher efficiency and cost-effectiveness. Interestingly, multiepitope vaccines can directly induce a T-cell immune response specific to the harbored epitopes while excluding the adverse effects of other epitopes in the intact antigen [34]. In order to examine the entire HPIV-3 proteome and create a multiepitope-based vaccination, we opted to use a variety of immunoinformatics and structural bioinformatics methods in the current study (Fig. 1). Initially, three biophysically stable proteins were chosen for prediction of B-cell and T-cell interaction and IFN-γ- and IL-4-producing epitopes from among all the HPIV-3 protein sequences that were made available in the database. Then, epitopes that have passed all of our selection parameters along with a proper adjuvant were joined in a well-organized manner into a multiepitope-based peptide vaccine (MEBV) that was assembled. To ensure the highest level of expression, stability, safety, and efficacy, the developed peptide vaccine was put through a battery of immunoinformatics tests. Although more accurate clinical testing is needed to confirm these findings, this research provides encouraging preliminary evidence that the HPIV-3 multiepitope vaccine may be able to elicit a robust immune response by interacting with human immune receptors.

## Methods

### Retrieval of protein sequences of HPIV-3

We underwent a thorough literature, and proteome analysis of HPIV-3 used the National Center for Biotechnology Information (NCBI) and the Bacterial and Viral Bioinformatics Resource Center (BV-BRC) server. The whole viral proteome of HPIV-3 (GenBank Accession: KF530252), including structural and non-structural proteins, was extracted in the first phase in FASTA format from the protein database.

### Analysis of physicochemical properties of the HPIV-3 proteins and antigenicity screening

The Expasy ProtParam tool (https://web.expasy.org/protparam/) was used to examine the physicochemical properties of all of the structural and non-structural HPIV-3 proteins [22]. The ProtParam tool was used to filter out proteins that would not be stable under in vitro conditions. The immunogenicity of the chosen proteins was predicted by the VaxiJen v2.0 server (http://www.ddg-pharmfac.net/vaxijen/VaxiJen/VaxiJen.html), with a threshold for likely antigen set at 0.4 [17]. SOPMA is a secondary structure analysis method that was used to forecast the four possible conformations of these proteins (helix, sheet, turn, and coils) [12]. When making predictions about the secondary structures of target proteins, we did not make any adjustments to factors like the number of conformational states, the similarity threshold, or the window width.

### T- and B-cell epitope prediction

As mentioned in the previous section, only the stable proteins predicted by the Expasy ProtParam tool were considered for their T-cell and B-cell epitope prediction [22]. Three predicted stable proteins were namely matrix protein (protein ID: AGT75284.1), hemagglutinin-neuraminidase (protein ID: AGT75286.1), and RNA-directed RNA polymerase L (protein ID: AGT75287.1). In an effort to reduce the total number of epitopes from 2233 amino acids to a more manageable size, epitope prediction was restricted to only the conserved sequence of RNA-directed RNA polymerase L (peptides: 388–604).

#### Cytotoxic T lymphocyte epitopes

The class of MHC molecule as well as the propensity at which an antigen epitope binds with MHC mostly determines the nature of immune response. Epitopes presented by MHC class I molecules are usually encountered by cytotoxic T cells. Therefore, cytotoxic T-cell epitopes of all three selected proteins were predicted by utilizing the “MHC-I binding” tool of the Immune Epitope Database (IEDB) server (http://tools.iedb.org/mhci/) [20]. In this case, we went for the default prediction method as “IEDB recommended 2020.04 (NetMHCpan EL 4.0)” [39] of “2013–02-22” version. This method takes a combinatorial approach to predict cytotoxic T-cell epitopes based on binding affinity with a reference set of human leukocyte antigen (HLA) alleles with population coverage [20].

The protein sequences were pasted in the box individually and submitted to obtain results. Other input parameters used were as follows: MHC source species (“human”), MHC allele (“Select HLA allele reference set”), and unchecked (“Show only frequently occurring alleles”). Alleles recognizing epitopes of nine amino acids long (nine-mer) were kept, and alleles recognizing the length of ten amino acids were discarded. The peptide epitopes were received as sorted by a descending order of prediction score. We only selected those cytotoxic T lymphocyte (CTL) epitopes with a better binding score and have interaction with a maximum number of HLA alleles.

#### Helper T lymphocyte epitopes

Then, we explored the “MHC-II binding” tool of the IEDB server to generate helper T-cell epitopes. The prediction method chosen was “IEDB recommended 2.2.” “Human, HLA-DR” was selected for species/locus [82]. This tool predicts MHC-II binding peptides according to percentile rank through comparing peptide sets selected randomly within the SWISS-PROT database against a submitted reference allele set [81]. In this study, “Select full HLA reference set” was selected to predict the helper T lymphocyte (HTL) epitopes exhibiting the most intense interaction with HLA alleles [60]. The peptide length was kept 15 amino acids long (15-mer). Individual protein sequences of FASTA format were pasted in the box and submitted to obtain results. In response, we obtained peptide sequences that had been ranked on the basis of percentile score, i.e., “Consensus” where a lower rank value indicates higher binding affinity.

#### B-cell epitopes

Since an effective humoral immune response comes through recognition of B-cell epitopes followed by antibody release, hence, B-cell epitopes are integral parts of a MEBV. We further took advantage of the “Antigen sequence properties” tool in B-cell epitope prediction menu in the IEDB server (http://tools.iedb.org/bcell/) for linear epitope prediction that can generate antibody response. The prediction method used was “Bepipred Linear Epitope Prediction 2.0” [36]. The protein sequences were pasted in the box individually and submitted to obtain results. Following the submission of the amino acid sequence in FASTA format in the submission box, we were given B-cell epitopes ranging in length from 10 to 60 amino acids. These particular peptides were chosen for the process of vaccine development.

### Epitope screening

#### Antigenicity

The VaxiJen v2.0 server (http://www.ddg-pharmfac.net/vaxijen/) was used to determine the antigenicity of the epitopes [18]. The epitopes were pasted individually, and all the parameters were kept as default.

#### Allergenicity

Possible allergenicity of the selected epitopes was checked through the AllerTOP 2.0 server (https://www.ddg-pharmfac.net/AllerTOP/) [15]. The epitopes were pasted individually, keeping all other parameters as default.

#### Toxicity

The epitopes of a vaccine candidate should be well tolerated once it is administered and non-toxic. There are several online tools available for assessing the toxicity of an epitope. We used the ToxinPred server (https://webs.iiitd.edu.in/raghava/toxinpred/multi_submit.php) to assess the toxicity status of the epitopes considered in this study [28]. Like other processes above, the epitopes were pasted individually in FASTA format and all the options were kept as default.

#### Human homology

To exclude any possibility of an autoimmune response/unresponsiveness, the epitopes were checked for sequence similarity against Human. As a publicly available online sequence alignment tool of NCBI, the BLASTP server (https://blast.ncbi.nlm.nih.gov/Blast.cgi?PAGE=Proteins) was used to determine the human homology of the epitopes. The epitopes were submitted individually in FASTA format, against “Homo sapiens (taxid:9606)” as organism, keeping all other options as default. In the results, epitopes with the *E* value greater than 0.05 were considered as non-homolog.

#### IFN-γ, IL-4, and IL-10 activation possibility

Helper T cells, after being stimulated by HTL epitopes, activate cytotoxic T cells and other immune effector cells by releasing a number of interacting cytokines like IFN-γ, IL-4, and IL-10 [49, 55]. Therefore, we evaluated all of our initially selected epitopes for their capability to induce these immune-regulatory cytokines. IFN-γ is a unique cytokine that has its role in both innate and adaptive antiviral CTL and HTL responses [13]. In this study, the IFNepitope server (http://crdd.osdd.net/raghava/ifnepitope/predict.php) was used to determine whether the selected epitope is a IFN-γ inducer or not [14]. The IL4pred server (https://webs.iiitd.edu.in/raghava/il4pred/design.php) and IL-10Pred server (https://webs.iiitd.edu.in/raghava/il10pred/predict3.php) were used to forecast IL-4 and IL-10 induction capability. The epitopes were pasted individually in FASTA format, and all the options were kept as default.

#### Human population coverage and epitope conservancy analysis

The vaccinations that are currently under development ought to be able to protect a sizable proportion of the populace of the entire world. In addition, the extent of the variation of HLAs differs considerably among populations. The IEDB (http://tools.iedb.org/population/) was used to assess 23 HLA class I and class II alleles examined in this study for their distribution across the world’s human population [7]. In the settings, “world” was selected as the region and “Class I and II combined” as the method of calculation. Again, the selected epitopes of a MEBV should represent very high conservancy among many reported isolates of the targeted virus (HPIV-3) around the world in order to rule out any cross-recognition which could lead to more complex immunological outcomes. Hence, we employed the default seven-allele method of the IEDB resource to determine the conservancy of the selected epitopes [60].

### Selection of epitopes for vaccine construction

We put forward a comprehensive and rigorous set of criteria for an epitope to be considered as an ideal candidate in the final vaccine construct. The epitopes which were an antigenic, non-allergenic, non-toxic, non-human homolog and which can induce at least one of the three cytokines (IFN-γ, IL-4, and IL-10) were selected eligible for vaccine construction.

### Immunization formulation: protein adjuvant and linker selection

Recombinant proteins are usually less immunogenic in nature. In this context, the use of adjuvants improves the efficiency of the both antibody-mediated and cellular immune responses to a recombinant protein vaccine by facilitating its absorption by antigen-presenting cells (APCs) [4]. In order to construct an effective multiepitope-based vaccination, it is necessary to unite anticipated epitopes with putative linkers in an efficient manner. AYY, GPGPG, and EAAAK are the linkers that are utilized the most frequently. Their primary application is to reduce junctional immunogenicity and increase pathogen-specific immunity [79]. We initiated our vaccine construct with linker “EAAAK” followed by an adjuvant which is “Beta-defensin 3” (UniProtKB ID: Q5U7J2_HUMAN). The epitopes that bind to MHC-I were connected using the linker “AAY,” the epitopes that bind to MHC-II were connected using the linker “GPGPG,” and the B-cell epitopes were connected using the linker “KK.” We ordered the eligible MHC-I, MHC-II, and B-cell epitopes of Matrix protein, Hemagglutinin-neuraminidase, and RNA-directed RNA polymerase L based on their chronological appearance in the sequence.

### Physicochemical properties of the vaccine peptide

ProtParam tool of ExPASy server (https://web.expasy.org/protparam/) computed the physicochemical characteristics of our designed vaccine polypeptide [80]. A number of well-studied physicochemical attributes including theoretical pI, grand average of hydropathicity, stability profiling, instability index, half-life, and aliphatic index were predicted by the ProtParam tool depending on the p*K*_a_ calculations of amino acids involved [6]. In addition to that, the solubility was analyzed using the SoluProt server version 1.0 (https://loschmidt.chemi.muni.cz/soluprot/). SoluProt is available both as a web program and as standalone software that uses gradient boosting machines to forecast the production of soluble proteins in *Escherichia coli* by taking some consensus 96-sequence-based characteristics, e.g., amino acid composition, sequence similarity to PDB sequences, and a number of aggregated physicochemical properties [53]. With an AUC of 0.62 and an accuracy of 58.5%, SoluProt outperforms other computing techniques.

### Prediction of antigenicity and allergenicity

It is important that the prospective vaccine, like the individual epitopes, poses no threat of allergenicity. Therefore, we used the AllerTOP 2.0 server to determine if our vaccination polypeptide was potentially allergenic. On the other hand, the antigenicity of the designed vaccine was predicted with VaxiJen v2.0.

### Secondary structure prediction of vaccine constructs

To foretell the vaccine construct’s secondary structure, we used the SOPMA program [23]. The amino acid sequence of the vaccine peptide was used as an input, and the output width was set to 70. Other common parameters for the number of conformational states our vaccine protein can take like similarity threshold and window width were each set to 4, 8, and 17, respectively (helix, sheet, turn, coil).

### Prediction and improvement of vaccine construct’s 3D structure

By running a streamlined version of AlphaFold v2.1.0 through the Colab notebook, we were able to make protein structure predictions [38]. After homology modeling, the GalaxyRefine server was used to correct any distortions in the returned protein structure [32]. After modeling and refinement, the structures were sent to a server called PROCHECK, which performed an analysis of the overall quality of the constructed and refined version of the vaccine candidate using a plot called Ramachandran plot. Following each in silico procedure, the Chimera 1.10.1 system provided us with assistance for visualization.

### Linear and conformational B epitopes of the vaccine

Without adjusting any prediction parameters, linear/continuous and conformational/discontinuous epitopes to be recognized by B cell receptors (BCRs) were predicted using ElliPro servers in the vaccine model [63]. B-cell epitopes, which can boost immunogenic reactions, should be present in the vaccine formulation for it to be successful as a preventative regimen.

### Molecular docking of Toll-like receptors with MEBV candidate

Human Toll-like receptors (TLRs), including TLR3, TLR4, and TLR8, with three-dimensional structures were acquired from the Protein Data Bank (PDB ID: 2a0z, 2z63, and 6zjz, respectively). Model refining of the receptor was performed using the GalaxyRefine server, after which any ligands that had been linked to the retrieved structures were deleted/excluded [32]. To examine the vaccine-TLR interaction profile, we used the ClusPro v.2 protein–protein docking server to conduct molecular docking analysis [43]. By minimizing the pairwise root-mean-square deviation (RMSD) energy between conformations, the server generates cluster scores based on rigid docking. The final model/version of the vaccine-TLR complex was chosen based on the lowest energy weight score and its members; this model was then visualized using the Chimera 1.10.1 system [62].

### Normal mode analysis (NMA) analyses using iMOD server

In order to examine and analyze the cumulative flexibility and mobility functions of the produced epitope vaccine in connection to the bound hTLR3, hTLR4, and hTLR8 protein target, the iMOD server was used due to its availability, speed, and efficiency [79]. This server can predict the overall stability of a docked complex on the basis of several indexes such as eigenvalues. Generally, with a higher eigenvalue, relatively harder deformation is expected [47].

### Clone optimization and creation in computer simulation

Easy and efficient expression of a recombinant vaccine in a bacterial system is one of the most important prerequisites while designing future candidate vaccines. The best suited candidate structure revealed through the immunoinformatic approach up to this point was subjected to undergo reverse translation and codon optimization in K16 host strain of *E. coli* through Java Codon Adaptation Tool (JCat) (http://www.jcat.de/Start.jsp) [25]. Codon optimization is a widely used technique that employs prokaryotic codon bias to significantly increase protein synthesis in the host expression system. While optimizing codons for *E. coli*, additional contributing factors like excluding rho-independent transcription terminators, binding sites for prokaryotic ribosome loading, and specific restriction sites for endonuclease cleavage were also taken under consideration. Codon adaptation index (CAI) value and GC content of the modified sequence are JCat’s final results. Excellent scores on the CAI ranging from 0.8 to 1.0 and GC content scores of 30 to 70% are typical.

Consequently, we took advantage of the SnapGene tool (www.snapgene.com) to clone the optimized nucleotide sequence of the final recombinant vaccine construct in the *E. coli* pET-32a + vector after introducing KpnI and BssSI restriction sites to the N- and C-terminals of the sequence, respectively.

### Experimenting the immune system virtually

Further analysis of the recombinant protein’s immunogenicity and corresponding immune response profile was performed using the C-ImmSim server (https://kraken.iac.rm.cnr.it/C-IMMSIM/index.php?page=1) [66]. C-ImmSim is an online simulation platform that uses multiple machine learning methods, including PSSM, to make predictions about immunological interactions. When the FASTA file containing the amino acid sequence was uploaded, the simulations began with the initial parameters found as follows: random seed = 12,345, simulation volume = 10, and simulation steps = 100.

### Molecular dynamics simulation of protein–protein interaction

The molecular dynamics (MD) program is useful for understanding how ligands and receptors behave when they produce complexes upon the docking procedure [29]. In this work, we used MD simulations to better understand the movement and stabilization of a protein-vaccine ensemble. The chosen cluster obtained during the molecular docking step, indicating the protein-vaccine complex, was utilized as the starting data configuration for the MD simulation [73]. The chosen cluster obtained during the molecular docking step, indicating the protein-vaccine complex, was utilized as the starting data configuration for the MD simulation in Gromacs 2023.1 version [64]. The force field parameters for the protein and vaccine complex were created using the CHARMM force field, providing plausible modeling of their interactions [29]. Sodium and chlorine ions were introduced to the solution to resolve the surface charge of the structure. For the simulation, a 10-angstrom-thick cage of TIP3P water atoms surrounded the protein-vaccine system [29]. By employing the steepest descent method, we minimized the energy of the structure by excluding steric collisions and decreasing the strength of van der Waals connections between the structure and water. To bring the entire system to its final state of equilibrium at 300 K and 1 atm, a 200-ps constant-volume (NVT) heating cycle was followed by a constant-pressure (MPT) equilibration [30]. Particle mesh Ewald (PME) strategies were used, employing a threshold of 10 angstroms, to determine the strength of non-bonded connections among molecules. The MD trajectory was subjected to several studies, including the RMSD, root-mean-square fluctuation (RMSF), the number of hydrogen bonds present, and the solvent-accessible surface area (SASA).

## Results

### Selection of protein targets to predict epitopes

The HPIV-3 proteome (with the accession number KF530252) consists of six structural and four non-structural proteins provided in Supplementary file 1. All eight HPIV-3 proteins and their in silico physicochemical properties received from the Expasy ProtParam tool are listed in Table 1.

**Table 1:** Physicochemical properties of the HPIV-3 proteins analyzed through the ProtParam tool

**Proteins**	**Molecular weight**	**Theoretical pI**	**Instability index**	**Stability profiling**	**Aliphatic index**	**Grand average of hydropathicity**
Nucleoprotein (515 aa)	57,786.07	5.21	45.31	**Unstable**	83.38	− 0.428
Phosphoprotein (602 aa)	67,433.36	5.55	47.56	**Unstable**	63.95	− 1.051
D protein (373 aa)	41,398.69	5.30	57.75	**Unstable**	46.54	− 1.323
C protein (199)	23,297.94	9.71	56.14	**Unstable**	92.56	− 0.700
Fusion glycoprotein F0	60,034.26	6.42	40.87	**Unstable**	109.94	− 0.003
Matrix protein (353 aa)	39,530.30	9.59	38.75	Stable	100.74	− 0.070
Hemagglutinin-neuraminidase	64,159.07	8.09	30.61	Stable	92.48	− 0.263
RNA-directed RNA polymerase L (2233)	255,944.92	6.12	36.99	Stable	98.28	− 0.243

According to the ProtParam tool, only three proteins were deemed to be stable, including matrix protein (antigenic value = 0.5857), hemagglutinin-neuraminidase (antigenic value = 0.5726), and RNA-directed RNA polymerase (antigenic value = 0.4556) L. Generally, unstable proteins are readily degraded by the host defense system and are not considered ideal for designing recombinant therapeutics. Moreover, unstable proteins with a high instability index are difficult to work with in vitro [41, 42]. Hence, we selected only these three stable proteins to design a potential vaccine against HPIV-3. All of the selected proteins displayed considerable variations in alpha helices, extended strands, beta turns, and random coils in their projected secondary structures (Table S2).

### Epitope filtering

Tables 2, 3, and 4 show in a visual way all the important features of the epitopes of the three proteins chosen for vaccine development. In a nutshell, vaccine-eligible epitopes should be antigenic, non-allergenic, and non-toxic; able to induce at least one of the three cytokines (IFN-γ, IL-4, and IL-10); and non-human homologous.

**Table 2:** The pivotal properties of matrix protein epitopes for vaccine development

Epitopes	Antigenicity	Allergenicity	Toxicity	Human homology	IFN-δ	IL-4	IL-10
**MHC-I**							
LPLDRSIKF	Antigen	Non-allergen	Non-toxic	Non-homolog	Non-inducer	Inducer	Non-inducer
SENGHIEPL	Antigen	Non-allergen	Non-toxic	Non-homolog	Inducer	Inducer	Non-inducer
GSLPIGLAK	Antigen	Non-allergen	Non-toxic	Non-homolog	Inducer	Inducer	Non-inducer
**MHC-II**							
VFLLGFFEMERIKDK	Antigen	Non-allergen	Non-toxic	Non-homolog	Inducer	Inducer	Inducer
GEFRYYPNIIAKGVG	Antigen	Non-allergen	Non-toxic	Non-homolog	Non-inducer	Inducer	Non-inducer
PSLPGEFRYYPNIIA	Antigen	Non-allergen	Non-toxic	Non-homolog	Inducer	Inducer	Non-inducer
EFRYYPNIIAKGVGK	Antigen	Non-allergen	Non-toxic	Non-homolog	Non-inducer	Inducer	Non-inducer

**Table 3:** The pivotal properties of hemagglutinin-neuraminidase epitopes for vaccine development

Epitopes	Antigenicity	Allergenicity	Toxicity	Human homology	IFN-δ	IL-4	IL-10
**MHC-I**							
IPISLTQQI	Antigen	Non-allergen	Non-toxic	Non-homolog	Non-inducer	Inducer	Non-inducer
**MHC-II**							
TYILWTITLVLLSIV	Antigen	Non-allergen	Non-toxic	Non-homolog	Non-inducer	Inducer	Inducer
SIVFIIVLTNSIKSE	Antigen	Non-allergen	Non-toxic	Non-homolog	Inducer	Inducer	Non-inducer
YILWTITLVLLSIVF	Antigen	Non-allergen	Non-toxic	Non-homolog	Non-inducer	Inducer	Inducer
**B cell**							
KARESLLQDINNEFMEVTEKIQVASDNTNDL	Antigen	Non-allergen	Non-toxic	Non-homolog	N/A	Inducer	Inducer
KVDERSDYASSGI	Antigen	Non-allergen	Non-toxic	Non-homolog	Non-inducer	Inducer	Non-inducer
EHPINENAICNTTGCPGKTQRDCNQASHSPWFSD	Antigen	Non-allergen	Non-toxic	Non-homolog	N/A	Inducer	Inducer
DYSDIRIKWTWHNVLSRPGNNECPWGHSC	Antigen	Non-allergen	Non-toxic	Non-homolog	Inducer	Inducer	Inducer

**Table 4:** The pivotal properties of RNA-directed RNA polymerase L epitopes for vaccine development

Epitopes	Antigenicity	Allergenicity	Toxicity	Human homology	IFN-δ	IL-4	IL-10
**MHC-I**							
ISISGVPRY	Antigen	Non-allergen	Non-toxic	Non-homolog	Inducer	Inducer	Non-inducer
**MHC-II**							
GEIELLKRLTTISIS	Antigen	Non-allergen	Non-toxic	Non-homolog	Non-inducer	Inducer	Inducer
YQSFIGIKFNKFIEP	Antigen	Non-allergen	Non-toxic	Non-homolog	Non-inducer	Inducer	Non-inducer
FIGIKFNKFIEPQLD	Antigen	Non-allergen	Non-toxic	Non-homolog	Non-inducer	Inducer	Non-inducer
SFIGIKFNKFIEPQL	Antigen	Non-allergen	Non-toxic	Non-homolog	Non-inducer	Inducer	Non-inducer
**B cell**							
RERHGGQWPPVTLPDHAH	Antigen	Non-allergen	Non-toxic	Non-homolog	Inducer	Inducer	Inducer
SLKEKEIKQEG	Antigen	Non-allergen	Non-toxic	Non-homolog	Non-inducer	Inducer	Non-inducer

### Population coverage analysis and conservancy analysis

HLA molecules are highly polymorphic, with a number of allelic variants and variable expression frequencies in the worldwide population. Consequently, for better patient coverage of vaccine diagnostics, T-cell epitopes with multiple allelic interactions are preferred. The population coverage utility of the IEDB predicts the proportion of people in a population who react to the antigens that make up each vaccine and are projected, based on the population’s HLA allele frequencies, to bind to the MHC-I and MHC-II supertype alleles.

In our study, global human population coverage analysis revealed that T-cell epitopes combined with HLA-I and HLA-II could cover 99.26% of the human population (Table S7 and Fig. S1). Meanwhile, we found that among known HPIV-3 isolates, all of our target epitopes were highly conserved (Tables S3 and S4).

### Strategies for joining epitopes together in a multi-epitope based vaccine

Then, with all the selected epitopes ready for constructing the final vaccine polypeptide, we must join these epitopes in such an efficient and well-recognized way that prevents any junctional immunogenicity and maintains the individuality of each epitope. In this context, short peptide linkers are well known for their ability to exert those specific functions. Hence, ideally, we put a putative “EAAAK” linker sequence to the N-terminal of our vaccine construct, which is followed by an adjuvant (“beta-defensin 3”; UniProtKB ID: Q5U7J2_HUMAN). β-Defensins are a leucocyte-derived family of antimicrobial peptides involved in eliciting immune responses, hence can be used as adjuvants [42, 45]. Additionally, β-defensin can stimulate TLRs or CCR6 for inducing innate and adaptive immune responses [45]. A number of immunoinformatics-based vaccines were designed by using β-defensin as an adjuvant [2, 5, 16, 57] The MHC-I epitopes were linked by the linker AAY, the MHC-II epitopes were linked by the linker GPGPG, and B-cell epitopes were linked by the linker KK. We placed the eligible MHC-I, MHC-II, and B-cell epitopes of matrix protein, hemagglutinin-neuraminidase, and RNA-directed RNA polymerase L chronologically.

**Fig. 2:**
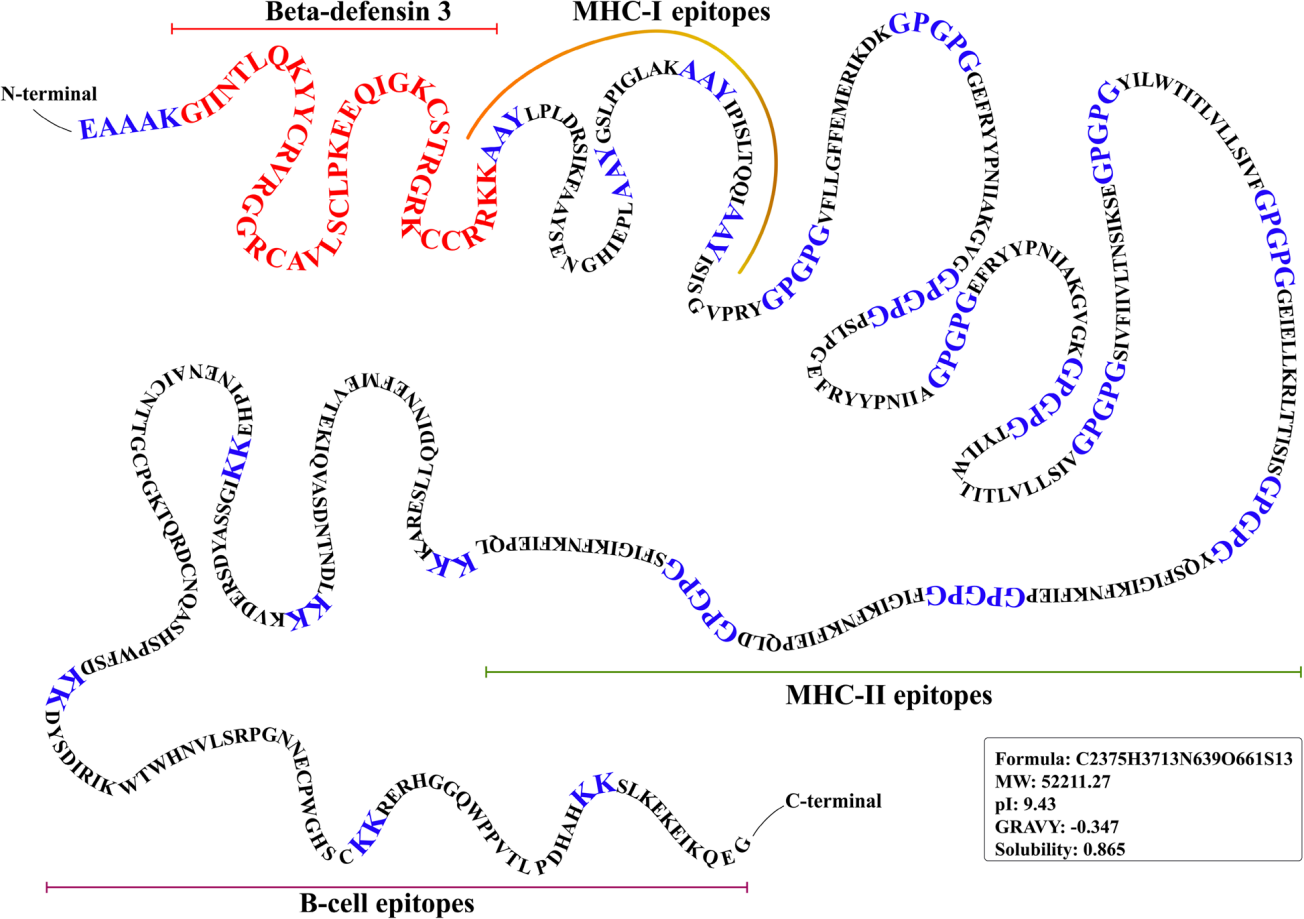
Final vaccine assemblage; MHC-I (CTL) = 5 epitopes, MHC-II (CD4 T) = 11 epitopes, B cell = 6 epitopes

The final product was of 478 amino acids long after the addition of linkers and adjuvants, with a total of 22 epitopes, including 5 CTL, 11 CD4 T cell, and 6 B-cell peptide sequences (Fig. 2).

### Physicochemical properties of vaccine peptide

According to physicochemical property calculations, our vaccine product is a 52-kDa protein (52,211.27 Ds) with a theoretical pI value of 9.43, which tells us that this protein is basic in nature. Combined, 41 negatively charged and 63 positively charged residues give our protein an estimated half-life of 1 h in mammalian reticulocytes, 30 min (in yeast, in vivo), and > 10 h (in *Escherichia coli*, in vivo). We determined the molecular formula of our protein as C2375H3713N639O661S13 with 7401 atoms. The instability index (II) is computed to be 35.62 and classifies the protein as stable. A protein with an instability index greater than 40 is unstable. The aliphatic index was estimated to be 80.61, indicating thermostability (the higher the aliphatic index, the more stable a protein is over a broad range of temperatures). Grand average of hydropathicity of GRAVY was the last physicochemical parameters to be obtained from this tool. Our vaccine protein received a GRAVY score of − 0.347, indicating that the protein is not hydrophobic. A solubility score of 0.865 indicates that this vaccine will be in soluble fraction during expression in a suitable host.

### Probable allergenicity and antigenicity of the vaccine peptide

The designed vaccine of our study, as an individual protein, is non-allergic, as demonstrated by the AllerTOP server. Furthermore, the VaxiJen v2.0 server predicted our vaccine protein to be sufficiently antigenic (score: 0.5561) to provoke an effective immune response with a threshold value of 0.4%. Therefore, this vaccine candidate is very promising for further structural and simulation studies.

### Secondary structure extrapolation of vaccine constructs

SOPMA can analyze the vaccine’s secondary structure by taking several parameters, like the conformational flexibilities and window widths, into consideration. SOPMA calculates and predicts the secondary structure of a given protein depending on the amino acid sequence of the protein. According to SOPMA calculations, among 478 amino acids, 92 amino acids are responsible for the helix formation (19.25%) and 120 for extended strands (25.10%), 35 are in beta turns (7.32%), and random coils are formed by 231 amino acids, which are major portions (48.33%) of the whole vaccine peptide (Fig. S2).

### Three-dimensional structure prediction and refinement

AlphaFold, the state-of-the-art AI system developed by DeepMind, was used to computationally predict protein structures with unparalleled accuracy and speed. It has enabled us to have the first computational method for accurately predicting protein structures with atomic level accuracy even in cases where no similar structure is known. Generally, AlphaFold has the power and flexibility to predict a protein structure that is most likely to appear as part of a PDB structure.

**Fig. 3:**
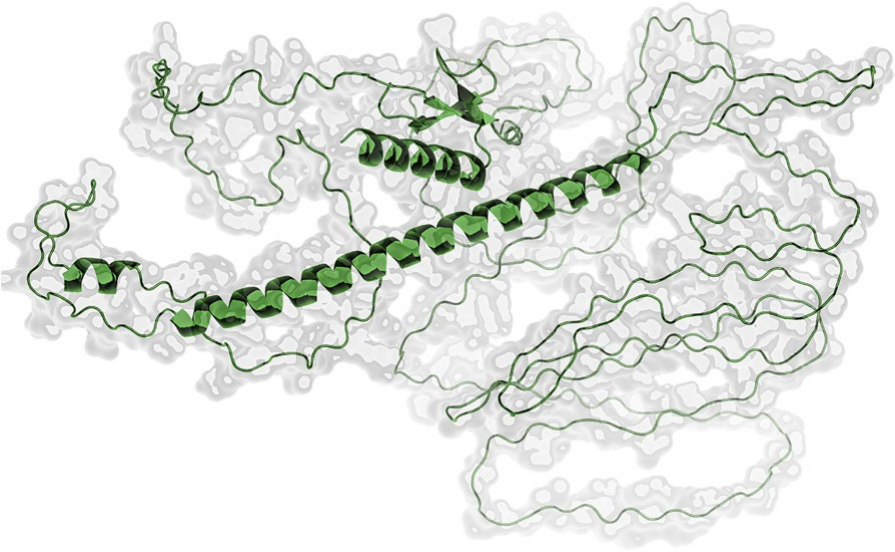
Vaccine construct’s final three-dimensional structure (cartoon model of helix and sheets)

**Fig. 4:**
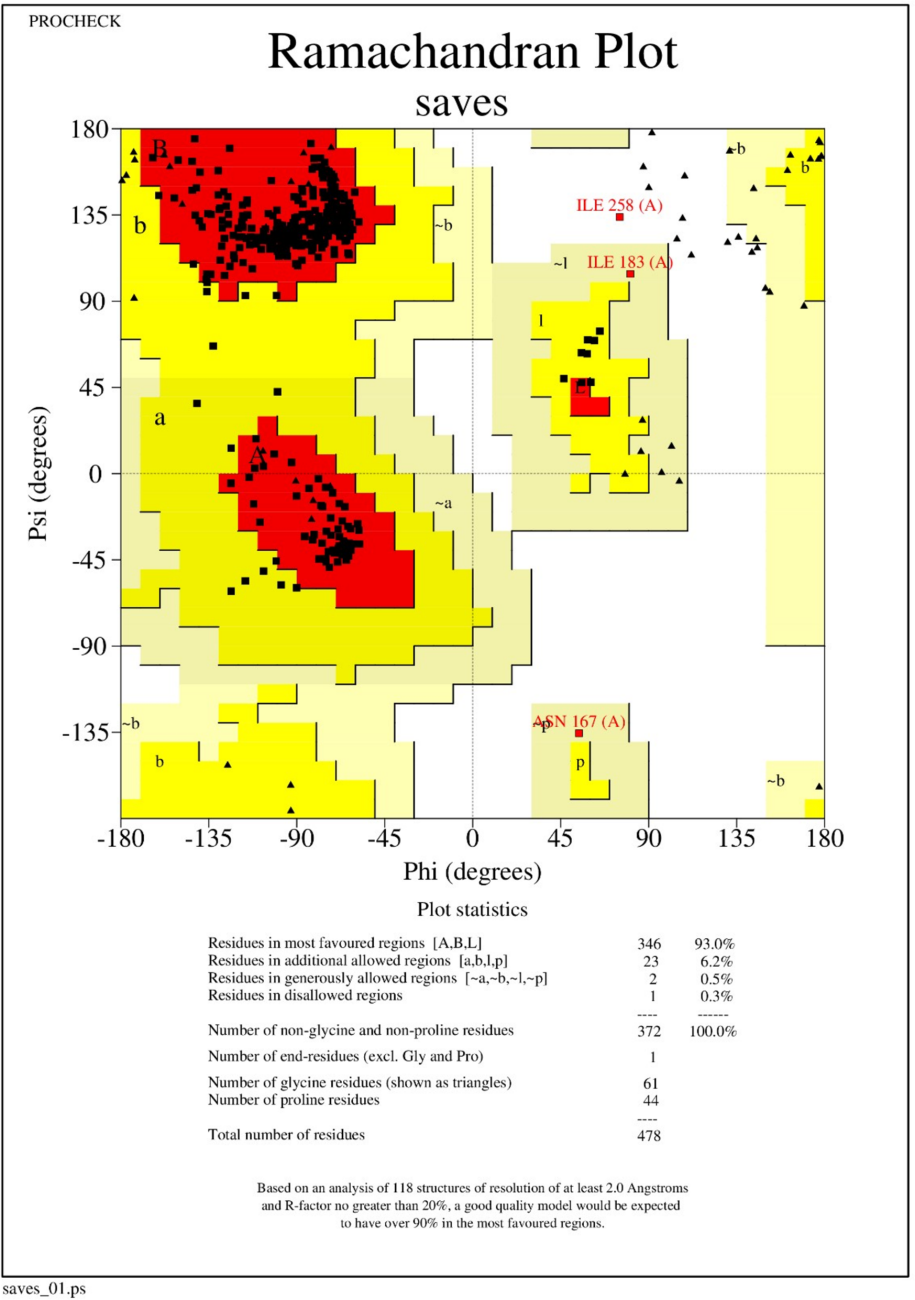
Ramachandran plot analysis of the vaccine structure after molecular refinement by the GalaxyRefine server. The GalaxyRefine server is presenting 93.00%, 6.2%, and 0.5% of the residues in favored, allowed, and only 0.3% disallowed regions

After we obtained the best 3D model from AlphaFold, the GalaxyWeb server refined and improved its structure quality through rigorous computations (Fig. 3). Consequently, among the five refined models generated by the server (Supplementary file 2), we compared initial and refined models from PROCHECK-generated Ramachandran plots. In conjunction to analyzing the residue geometry in the postscript plots, it checks the quality of structures solved by NMR. After the refinement, the model harbored 93.0%, 6.2%, 0.5%, and 0.3% of the residues in the most favored, the additional allowed, the generously allowed, and the disallowed regions each (Fig. 4). Besides, it also exhibited a pass signal to all-residue Ramachandran plots, all-residue chi1-chi2 plots, residue property plots, and RMS distances from the planarity plot (Supplementary file 3).

In the meantime, we further validated the predicted structure of the vaccine peptide by applying a program named ERRAT, which verifies protein structures determined by crystallography. ERRAT is lightly termed as “overall quality factor” for non-bonded atomic interactions, where a high score usually indicates a better quality. For instance, a value > 50 resembles a high-quality model. We received an ERRAT score of 95.238 (Supplementary file 3) for our vaccine, which indicates a very high level of accuracy for in silico protein structure determination.

### Linear and conformational B epitopes of the vaccine

The ElliPro server suggested nine linear/continuous epitopes (Table S5) with corresponding scores varying from 0.52 to 0.799, and the size of epitopes varied from 7 to 55 residues.

On the other hand, according to the server, all 478 residues of the vaccine protein were well distributed in 20 conformational B-cell epitopes, with a variable score starting from 0.50 to 0.818 (Table S6) and a number of differential epitope lengths (3 to 32 residues).

### Molecular docking of TLR3, TLR4, and TLR8 with MEBV Candidate

We were able to simulate and analyze the stable and dynamic interactions between the multiepitope vaccine (ligand) and three innate immune receptors using ClusPro 2.0 and a pictorial database called PDBsum.

**Fig. 5:**
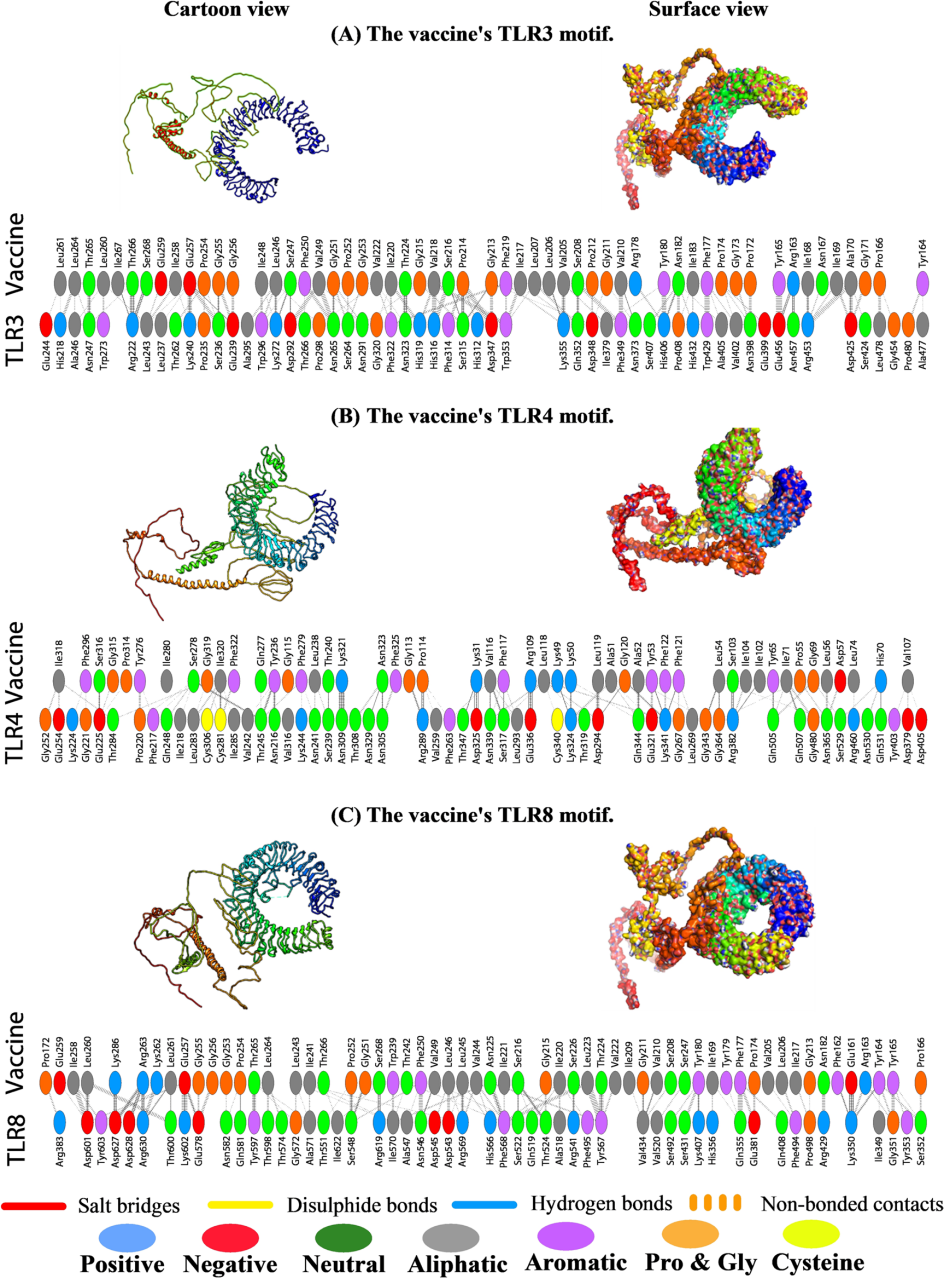
The individualized TLRs’ interaction pattern of the newly designed vaccine by means of the ClusPro docking server. **A** TLR3-MEBV complex interaction pattern, **B** TLR3-MEBV complex interaction pattern, **C** TLR8-MEBV complex interaction pattern

In total, ClusPro 2.0 generated 30 models (Supplementary file 4), and the model number 01 for TLR3, model number 02 for TLR4, and model number 03 for TLR8 exhibited the lowest binding energy score, − 1754.6 kcal/mol with 39 members for TLR3, − 1626.4 kcal/mol with 35 members for TLR4, and − 1708.9 kcal/mol with 25 members for TLR8, respectively, which implicate a good affinity and stability of the docked complexes (Fig. 5). As a result, a significant number of a well-defined molecular interaction has been projected between predicted vaccine construct with TLR3, TLR4, and TLR8 receptors. The interface statistics for TLR3, TLR4, and TLR8 receptors are represented in Table 5.

**Table 5:** The interface statistics for TLR3, TLR4, and TLR8 receptors after being docked with the MEBV

**Chain**	**No. of interface residues**	**Interface area (Å**^**2**^**)**	**No. of salt bridges**	**No. of disulfide bonds**	**No. of hydrogen bonds**	**No. of non-bonded contacts**
**Interface statistics (TLR3)**						
Receptor	56	2607	2	–	28	405
Vaccine	55	2670				
**Interface statistics (TLR4)**						
Receptor	57	2688	2	–	38	334
Vaccine	49	2969				
**Interface statistics (TLR8)**						
Receptor	52	2813	5	–	30	359
Vaccine	58	2718				

### Evaluation of normal mode analysis

**Fig. 6:**
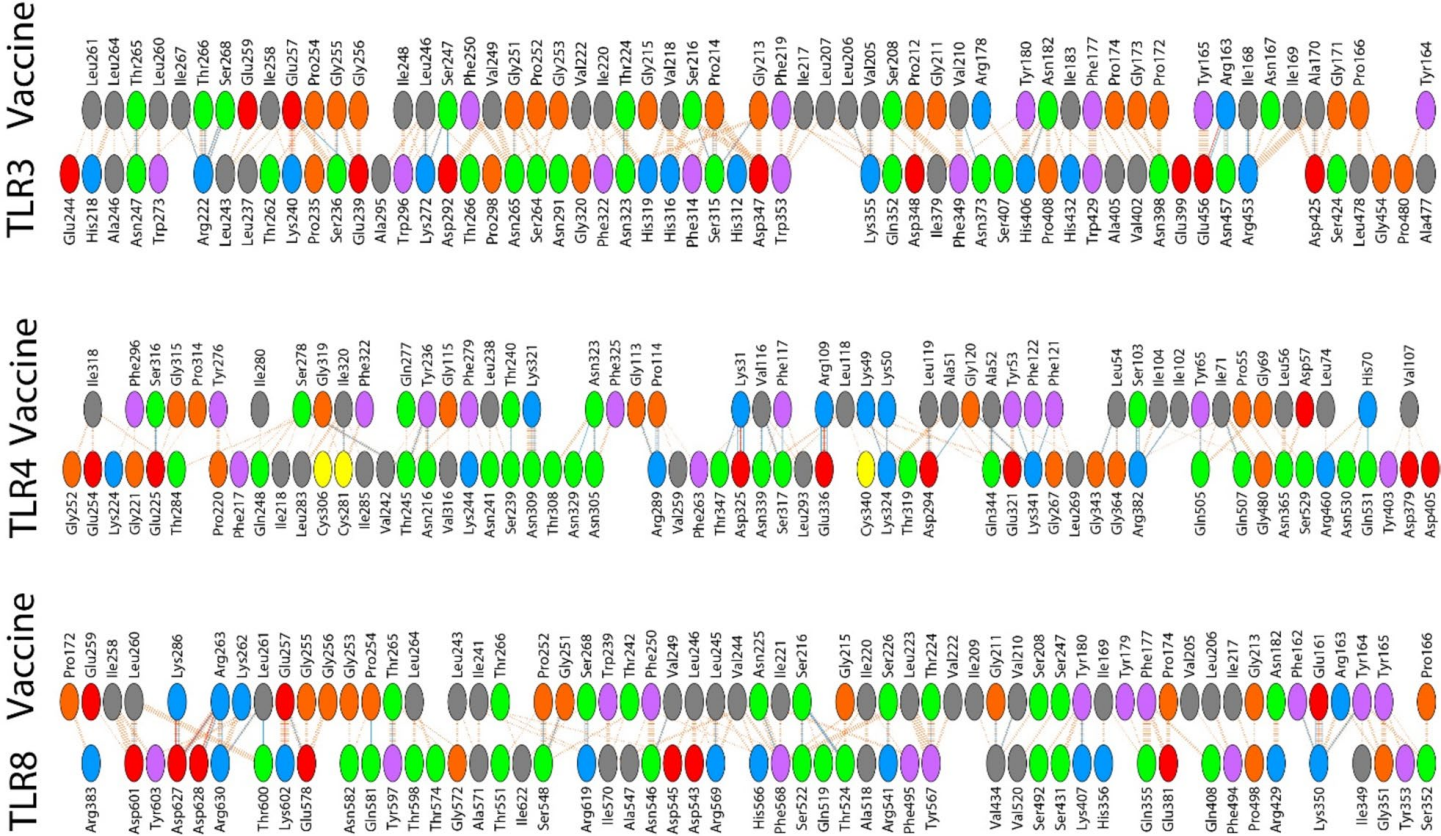
The outcomes of normal mode analysis of MEBV-hTLR3, MEBV-hTLR4, and MEBV-hTLR8 docked complexes

Publicly available online server IModS is widely used for structural analysis that involves making changes to the complex’s force field at different time intervals. As a result, the model that comes out of this usually has less distortion in all of the capacities that the residues represent. Eigenvalue calculations reveal that the MEBV-hTLR3 complex and MEBV-hTL4 has a value of 1.173938*e* − 07 and 1.210420*e* − 07, whereas the MEBV-hTLR8 complex has a value of 1.27641*e* − 07. A low RMSD and a highly co-related region in the heat maps indicated that the individual residues interacted more effectively with one another. A comprehensive description of the results of the IModS molecular dynamics simulations is provided in Fig. 6.

Our further exploration of the log file supplied by IModS specified that the sum total of dihedral angles for MEBV-hTLR3, MEBV-hTLR4, and MEBV-hTLR8 is 2220, 2024, and 2330, respectively. In addition, the sum of the rotational and translational ICs (non-Eckart) for both complexes, including MEBV-hTLR3 complex and MEBV-hTL4, was 12. On the other hand, the value was 36 for the MEBV-hTLR8 complex.

### In silico trial immune simulation

**Fig. 7:**
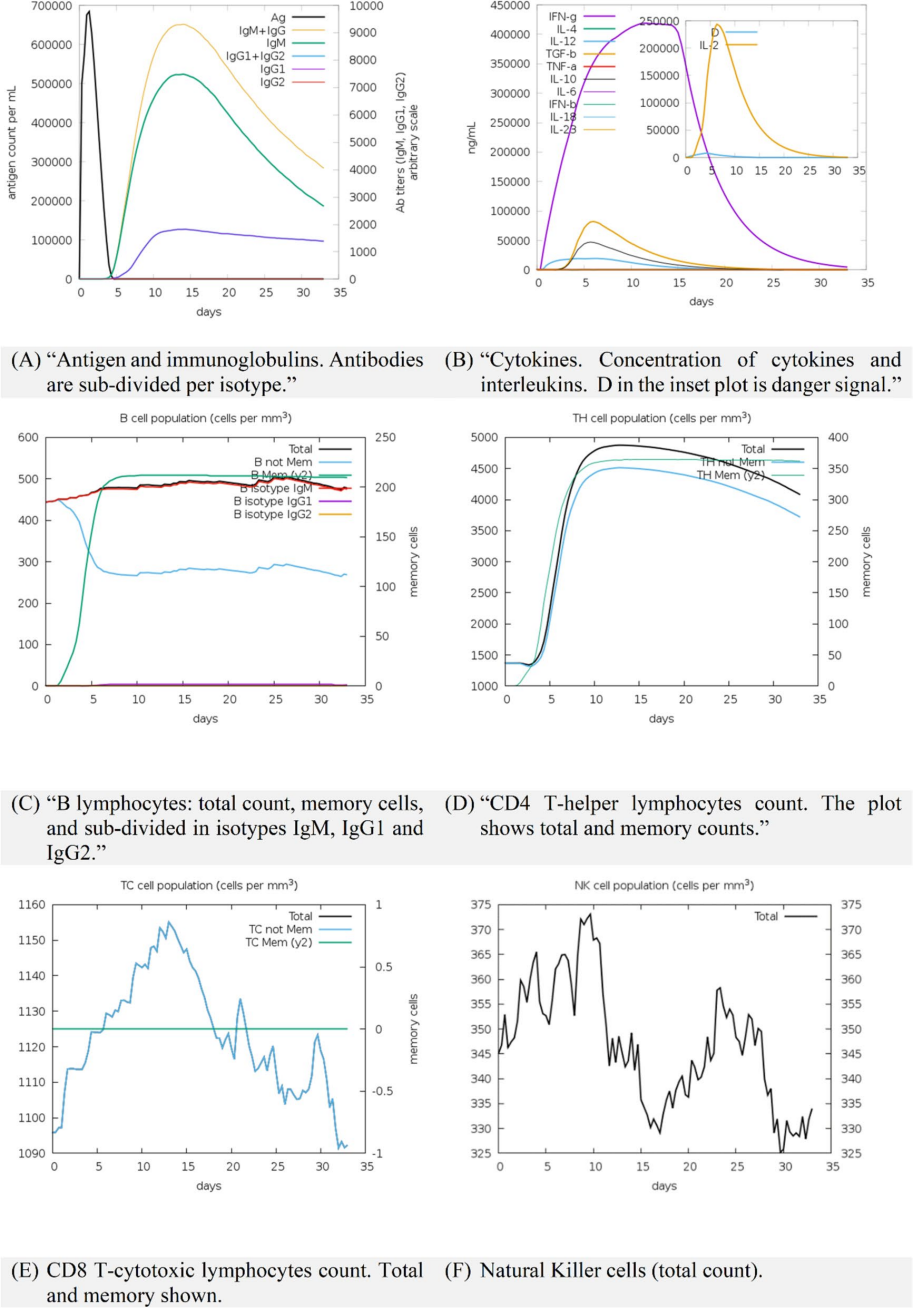
Modeling the immune system’s reaction to a simulated vaccine antigen via computer simulations (**A**–**F**)

We used the C-ImmSim platform to simulate the interaction between our vaccine protein and various components of the human immune system in silico. Here, in the simulation trial results, we observed the pattern of antibody response, secreted cytokine profile, and the dynamics of cellular response (lymphocytes and WBCs) (Fig. 7). Counts are plotted in units as in figure title or legend, and time is expressed in days.

As presented in Fig. 7, the secondary and tertiary antibody responses produced by the simulation were significantly higher than the primary response. The simulation results show that immunoglobulin (Ig) production can begin 3–5 days post immunization which can reach its highest level on 10–15 days depending on the dose (Fig. 7A). The results of the production of interleukins and cytokines indicate a strong response of pro-inflammatory cytokines such as IFN-γ and IL-2 with the target vaccine (Fig. 7B). The population of B cells, TH cells, and TC cells as well as the proliferation status of NK cells are shown in Figs. 7C through F, respectively. The simulation study also recorded a considerable rise in the number of B cell memory and active T cells. Collectively, these results demonstrate that our vaccine’s design undoubtedly has the potential to elicit an immune response and lay the groundwork for immunization against HPIV-3-related illnesses.

### In silico cloning and optimization

Appropriate Codon optimization can increase the expression profile of a recombinant protein in a host system. After running the codon optimization module in JAVA tool, we received a 1434-nucleotide-long transcript for 478 amino acids (Fig. S4). A CAI value of 0.927 for optimized nucleotide sequence, with 51.25% GC content, tells us an excellent possibility expression of the recombinant vaccine in the *E. coli* host.

**Fig. 8:**
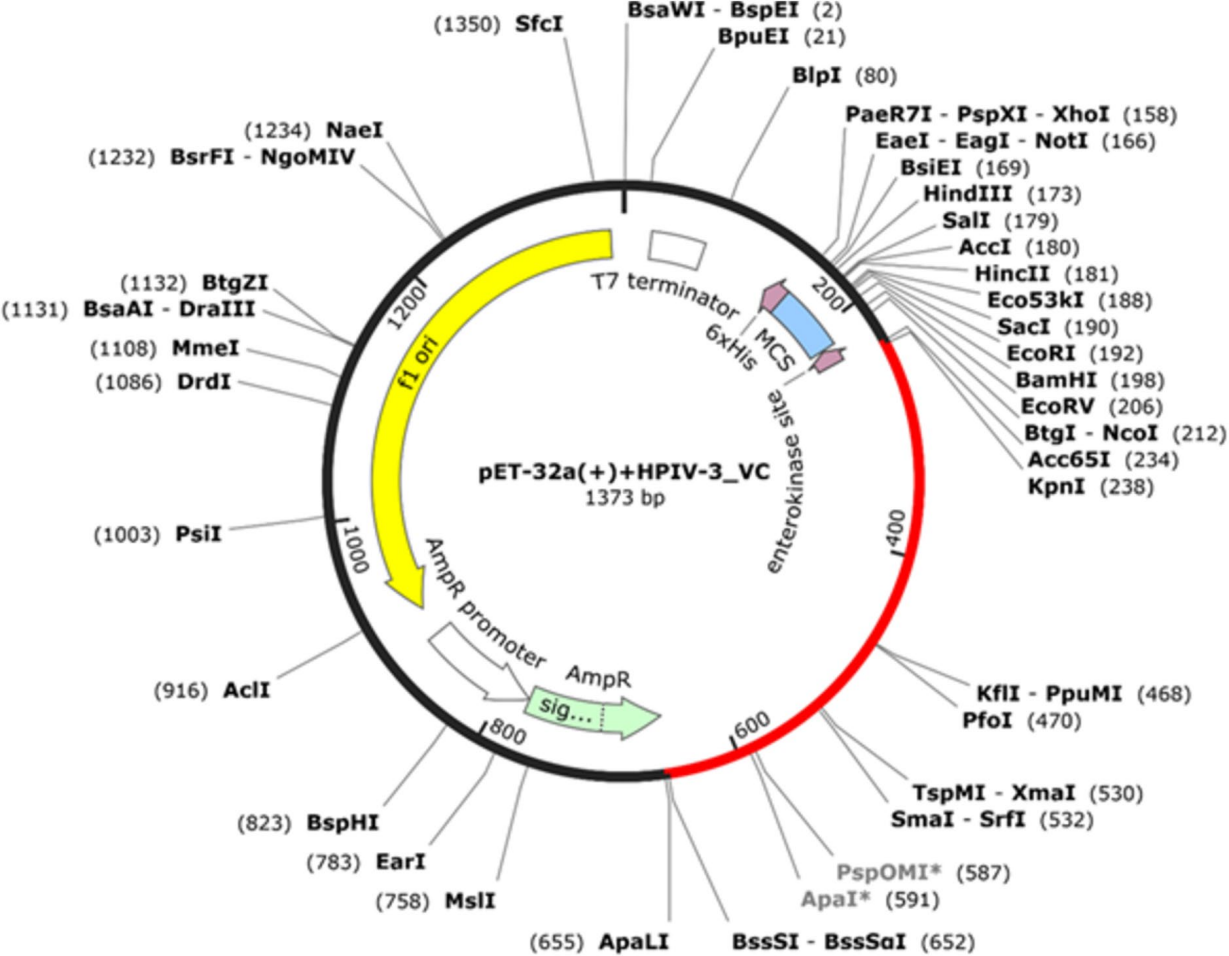
In silico cloning of MEBV into a pET-32 ( +) vector in flanked by KpnI and BssSI restriction positions with codon adaptation and enhancement for expression

Lastly, the SnapGene software platform assisted us to insert adapted codon sequences into a pET32a + vector by assisting KpnI and BssSI restriction enzymes. The final product (vector and optimized codon sequence) consists of 1373 bp (Fig. 8).

### MD simulation results of protein–protein interaction

**Fig. 9:**
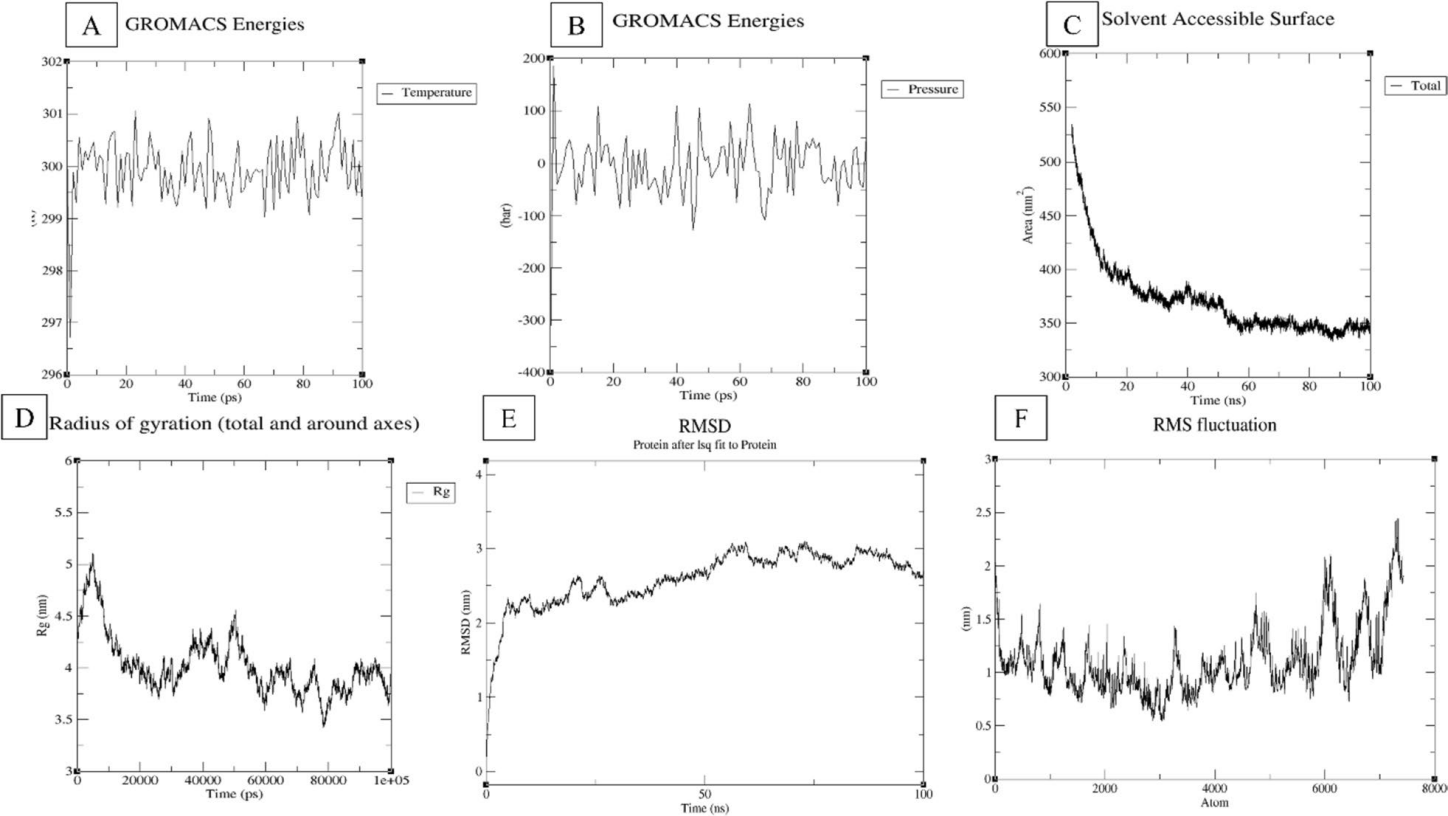
**A**–**F** Illustrations of MD simulation results of the modeled vaccine and TLR4 complex

The structural analyses of the vaccine-TLR4 complex from the Gromacs simulation represent the stability and interaction of the protein–protein complex. The pressure graph (Fig. 9A) showed an average temperature variation of 300 K during 100 ps while the pressure graph suggested an average temperature change of around − 3 bar (Fig. 9B). According to Fig. 9C, the average SASA value was 371.0 nm^2^, whereas, after 60 ps, the SASA value remained steady with an average of 346.0 nm^2^. The Rg (radius of gyration) plot (Fig. 9D) showed an average fluctuation of around 4.0 nm for 100 ps. Additional analysis employing RMSD revealed the structural stability of the protein–protein complex (human TLR4 and vaccine). The simulation of the complex structure showed an average RMSD fluctuation of 2.5 nm throughout the timeframe (Fig. 9E). Similarly, the fluctuation of amino acid residues and the RMSF was investigated. The highest fluctuations of the complex were observed in the N- and C-terminal portions with the RMSD value around 2 nm. Overall, the RMSF change was steady, fluctuating by an average of 1.1 nm during the time period.

## Discussion

The worldwide prevalence of HPIV-related hospitalizations and illnesses underpins the necessity of considering this virus as a possible threat to patients of all ages, not just newborns and the very young. Out of the four other well-recognized serotypes, it has recently been identified that HPIV-3 is the most virulent serotype and can cause severe sickness in Asian newborns, out of the four other well-recognized serotypes. Despite a drop in enveloped respirovirus prevalence during the COVID-19 pandemic, possibly due to social distancing and hygiene practices, a steady rise in HPIV infections has been documented with easing measures, particularly in Asian territories [41]. Vaccines should prevent such respiratory illness–related viruses in children, pregnant women, and the elderly. However, there are no licensed vaccines currently available for the prevention or treatment of HPIV infections [61]. In light of these circumstances, the purpose of this study was to develop a multiepitope vaccination against HPIV-3 by focusing on its three stable antigenic proteins (HN, MP, and RRP).

The application of vaccines has traditionally been regarded as the most efficient and affordable way to treat diseases caused by infectious pathogens. The current research sought to design a multiepitope vaccine, since MEBVs have several advantages over their traditional methods and platforms that use single-antigen counterparts. Because of (i) its myriad of HLA interactions, each of which is represented by a different hTCR, (ii) its multiple epitopes from different target proteins or antigens of the target disease, which increases the spectrum of the immune response against the prioritized virus, (iii) its flexibility to cover a massive community, and (iv) the fact that an MEBV harbors only immunogenic epitopes, it therefore provokes a long-lasting immune response selectively against these epitopes, which automatically excludes any probable unwanted immunogenicity [31, 37, 48, 69]. Furthermore, there are additional immunoengineering opportunities to improve immunogenicity by incorporating immune-boosting regimens [31, 37, 48, 69]. No wonder contemporary immunologists and vaccinologists are constantly seeking newer and more robust approaches that make use of bioinformatics platforms to create an efficient and viable MEBV. Recent advances in computational algorithms for prediction, design, and simulation of novel drug and vaccine candidates have significantly lowered the experimental workload by allowing us to deal with a vast amount of genomic, transcriptomic, and proteomic data within a short time [66].

According to the Centers for Disease Control and Prevention (CDC), USA, there are no licensed HPIV vaccines available for human application, although clinical trials are well underway (https://www.cdc.gov/parainfluenza/prevention-treatment.html). Previously, due to relatively high pathogenicity of HPIV-3 compared to other serotypes, several structure-guided approaches evaluated the neutralization potential of vaccines in animal models [71, 76]. However, after an extensive literature survey, we found the current study will be the first attempt to take an immunoinformatic approach to explore HPIV-3 proteome for a rational design of a MEBV. Essential to the success of a multiepitope vaccination is the meticulous selection of antigenic epitopes from the target proteins [3, 85]. Like all other enveloped viruses, HPIVs also consist of several structural and non-structural proteins [72]. Physicochemically unstable proteins are really difficult to work with in vitro, and the way an antigen is identified by B cells and T cells strongly influences immunity to that antigen [19, 70, 75]; the most stable antigenic proteins (HN, MP, and RRP) of HPIV-3 were used to generate an extensive set of B cell and T cell (MHC-I and MHC-II) libraries. Next, the best-fit epitopes were screened for inclusion in the multiepitope-based peptide construct in order to induce both innate and adaptive immunity when the vaccine construct was exposed. For selecting epitopes, a number of well-established immunological and vaccine features were taken into consideration, such as antigenicity, allergenicity, capability to induce IFN-γ or IL-4 or IL-10, conservation, multiple allelic affinity, sequence homology with known human proteins, wide population coverage, and effective molecular interaction with their respective HLA alleles [50, 59, 77].

In order to combine the chosen epitopes into a final vaccine peptide, we then chose putative linkers, including EAAAK, AAY, GPGPG, and KK, that effectively separate the epitopes. To further boost the immune response to this possible vaccine, additional components were added to the final vaccine formulation beyond only these epitopes. In the first place, EAAAK was used to boost the bi-functional enzymatic performance, provide structural strength, and stabilize the fusion protein [9]. Next, our team took AAY as a secondary linker for affixing CTL epitopes. AAY linkers were employed to decrease the number of β-turn and coil regions while increasing the number of α-regions; this helps to form epitopes in a more natural shape while preventing the production of junctional epitopes, which enhances the presentation of the epitope [26, 67]. As an added advantage, it has been speculated that the use of such linkers amplifies the expression of target proteins [67, 84]. As GPGPG may both disrupt the junctional immunogenicity and trigger an HTL immune response, it was chosen as the third linker. This allows the immunogenicity of specific epitopes to be restored [86]. The pH value was brought down to a more physiological range by using the KK linker, which was the last one used [26]. One of the fundamental drawbacks of peptide vaccines is their short duration of protection. By including a defensin adjuvant, we were able to deposit the antigenic molecule at the site of the vaccine, where it would be released slowly over time, thereby elongating the vigor of the immune response [27].

The final length of our constructed peptide vaccine is 478 amino acid residues with 52.21 MW, which is very close to the standard range of MEBV (40–50 kDa) [44]. Also, it was found that our vaccine design was sufficiently antigenic and safe in terms of allergenicity. The overall stability of the final vaccine construct was confirmed by a better instability index, a favorable theoretical pI, and a greater estimated half-life to generate a vigorous immune response. Furthermore, we took advantage of available in silico refinement and validation methods to verify the quality of our constructed vaccine model, which projected more than 90% of the residues to reside in the favored region of the Ramachandran plot. Overall, the resultant construct demonstrated promising physicochemical, immunological, and chemical properties when evaluated computationally, and molecular dynamics simulation studies were used to provide a detailed look at how this possible vaccine interacted with immune system receptors.

The TLRs are crucial components of the innate immune system due to their inherent ability to initiate an effective cytokine and inflammatory response against foreign particles by recognizing the molecular signatures more commonly known as PAMPs [1, 40]. Accordingly, vaccine-TLR molecular interaction should provide us with a more detailed picture of the vaccine’s potential to kick-start a series of signaling events that establish an antiviral state. Among the characterized TLRs of human origin, TLR1, TLR2, TLR3, TLR4, TLR6, TLR7, TLR8, and TLR9 have been reported to be involved in antiviral immune responses [78]. More precisely, TLR3, TLR4, and TLR8 have been found to recognize virions with ssRNA as genetic material [78]. In addition, HPIV-3 infection can result in dsRNAs, which can be readily recognized by TLR3 receptors [8, 33]. Hence, for this particular study, we selected TLR3, TLR4, and TLR8 for docking and molecular dynamics simulation against the designed multiepitope vaccine peptide by the ClusPro server. The docking analysis revealed that our vaccine model has sufficient affinity for TLR3, TLR4, and TLR8 so that they can act as sensors for recognizing HPIV-3 epitopes followed by the commencement of an immune response. Moreover, the stability of the docked complex was demonstrated by different indexes from iMOD server (the main-chain deformability plot, B-factor values, eigenvalue, covariance matrix, and elastic network model). Thus, we observed a clear reflection on the robustness of the engineered vaccine through computing its internal coordinates via NMA [47]. The greatest eigenvalue demonstrates the stability of our assembled complexes and indicates it will likely not deform under the stress of an immune response. In addition, the Gromacs simulation revealed that the RMSD and RMSF values of the human TLR4 and vaccination complex were consistent, resulting in improved stability and compactness between the complex’s constituent parts. Besides, the SASA and Rg values became lowered and stabilized with time, confirming the steadiness of the complex structure.

Our later in silico simulation of the immune response using the vaccine as an antigen covered major components of the adaptive and innate immune system (T cells, B cells, NK cells, antibodies, and cytokines) [66], and the simulation claimed that antibody and cytokine levels peak between 5 and 15 days after vaccination. Importantly, a significant rise in IgM + IgG levels was seen among the antibodies. Overall cytokine levels were higher, while INF cytokine levels were extraordinarily prevalent. The vaccine candidate also facilitates the generation of NK cells, cytotoxic T cells, helper T cells (CD4^+^), and memory B cells. Previously, Naveed et al. [58] reported that MEBV candidate–targeted hemagglutinin-neuraminidase protein increased the level of B cell, T cell (TH, TR, TC), and NK cell population against HPIV-1 in silico. Hence, these results demonstrate that our proposed vaccine possesses propensity to provoke both the innate and adaptive components of the immune system and most likely can counteract HPIV-3. As this is the case, we urge that professionals/clinicians perform experimental validation of the proposed vaccine to determine its tolerability, pharmacokinetics, and profitability.

In order to validate the efficacy of an engineered vaccine in terms of its immunoreactivity, we must express the vaccine peptide in a bacterial system in a cost-effective manner [24]. *E. coli* has been the preferred host (K12 strain) organism for expression of recombinant proteins of immunological/therapeutic interest [35, 68]. Furthermore, to facilitate optimum-level expression of the vaccine protein, the codon was adjusted according to the host organism (*E. coli*), which led to highly favorable transcript structure with a CAI value of 0.927 and a GC content of 51.25%. Nonetheless, robust experimental characterizations would be required to verify these computational forecasting and predictions.

HPIV-3 has been reported as the most pathogenic, especially in the Asian and South Asian regions, causing LTRI in children younger than 5 years of age [65, 74]. Although predicting the outbreak of a virus like HPIV-3 is really difficult, we can never rule out the possibility after a SARS-CoV-2 pandemic. In this context, with very few clinically tested therapeutic options and a non-existent vaccine could leave clinicians clueless about how to curb the disease in children. Hence, the model MEBV designed in this study against HPIV-3 using several in silico and immunoinformatic methods will be a resource for future experimental vaccinators who will need to go through the next stages of preclinical and clinical safety, efficacy trials, and finally, approval for mass immunization.

## Conclusion

Among the four types of HPIV, HPIV-3 is speculated to be the most pathogenic since it can infect both young children and the elderly who have compromised immune systems, resulting in life-threatening cases of acute lower respiratory disease. Any future HPIV-3 outbreak would be disastrous for global public health because antiviral medicines are scarce and vaccine candidates must be registered. In this research study, a MEBV was developed specifically for HPIV-3 using a systematic reverse vaccinology technique. Analysis of the vaccine candidate revealed that it was extremely stable, antigenic, non-allergenic, and non-toxic, suggesting that it had the potential to elicit strong cellular and humoral immune responses. Moreover, the proposed vaccine showed potent binding interactions with three common immune receptors, which are crucial in the innate immunological response of humans.

### Supplementary Information

**Additional file 1: Table S1. **Strain information. **Table S2.** Secondary structural properties of target proteins. **Table S3.** Most potential non-allergen, nontoxic, 05 T-cell epitopes with interacting MHC-I alleles, epitope conservancy score. **Table S4.** Most potential non-allergen, nontoxic, 11 T-cell epitopes with interacting MHC-I alleles, epitope conservancy score. **Table S5.** Predicted linear epitope(s): ElliPro. **Table S6.** Predicted discontinuous epitope(s): ElliPro. **Table S7.** Population coverage calculation result. **Fig. S1.** Human population coverage analysis. **Fig. S2.** Secondary structure prediction by SOPMA. **Fig. S3.** Conformational B-cell epitopes. **Fig. S4.** Cloning in pET-32 (+) vector after restriction site addition and in silico PCR amplification of the vaccine construct.

**Additional file 2.**

**Additional file 3.**

**Additional file 4.**

## Data Availability

All relevant data are within the manuscript and its Supporting Information files.
