# Supplementary material for: Immunoinformatics-aided rational design of multiepitope-based peptide vaccine (MEBV) targeting human parainfluenza virus 3 (HPIV-3) stable proteins
Source: J Genet Eng Biotechnol. 2023 Dec 6;21:162. doi: 10.1186/s43141-023-00623-5 (PMC10700276; doi:10.1186/s43141-023-00623-5)
Supplement: Supplementary file 1 — Additional file 1: Table S1. Strain information. Table S2. Secondary structural properties of target proteins. Table S3. Most potential non-allergen, nontoxic, 05 T-cell epitopes with interacting MHC-I alleles, epitope conservancy score. Table S4. Most potential non-allergen, nontoxic, 11 T-cell epitopes with interacting MHC-I alleles, epitope conservancy score. Table S5. Predicted linear epitope(s): ElliPro. Table S6. Predicted discontinuous epitope(s): ElliPro. Table S7. Population coverage calculation result. Fig. S1. Human population coverage analysis. Fig. S2. Secondary structure prediction by SOPMA. Fig. S3. Conformational B-cell epitopes. Fig. S4. Cloning in pET-32 (+) vector after restriction site addition and in silico PCR amplification of the vaccine construct. [file 43141_2023_623_MOESM1_ESM.docx]

Table S1: Strain Information

| Strain Name: | HPIV3/USA/629-D01363/2008 |
| --- | --- |
| Organism: | Human parainfluenza virus 3 |
| Taxonomy: | Paramyxoviridae -> Paramyxovirinae -> Respirovirus -> Human parainfluenza virus 3 |
| GenBank Host: | Homo sapiens |
| Isolation Country: | USA |
| GenBank Sequence Accession: | KF530252 |
| Sequence Length: | 15405 |
| Sequence Status: | Complete |
| Number of Proteins: | 8 |
| Sequence Status: | Complete |

Table S2. Secondary structural properties of target proteins

| **Structural Glycoprotein** | **Alpha Helix** | **3_10_ Helix** | **pI helix** | **Beta Bridge** | **Extended Strand** | **Beta Turn** | **Bend Region** | **Random Coil** | **Ambiguous States** | **Other states** |
| --- | --- | --- | --- | --- | --- | --- | --- | --- | --- | --- |
| Matrix protein | 19.55% | 0% | 0% | 0% | 24.08% | 5.67% | 0% | 50.71% | 0% | 0% |
| Hemagglutinin-neuraminidase | 18.71% | 0% | 0% | 0% | 26.92% | 3.67% | 0% | 50.70% | 0% | 0% |
| NA-directed RNA polymerase L | 50.07% | 0% | 0% | 0% | 10.17% | 3.67% | 0% | 36.09% | 0% | 0% |

| Allele seq_num | start | end | Length | Peptide | Score | Percentile rank | Conservancy |
| --- | --- | --- | --- | --- | --- | --- | --- |
| HLA-B*35:01 | 163 | 171 | 9 | LPLDRSIKF | 0.969928 | 0.02 | 100.00% |
| HLA-B*53:01 | 163 | 171 | 9 |  | 0.924114 | 0.02 |  |
| HLA-B*51:01 | 163 | 171 | 9 |  | 0.761172 | 0.05 |  |
| HLA-B*07:02 | 163 | 171 | 9 |  | 0.715535 | 0.12 |  |
| HLA-B*08:01 | 163 | 171 | 9 |  | 0.651452 | 0.08 |  |
| HLA-A*26:01 | 163 | 171 | 9 |  | 0.049612 | 1.2 |  |
| HLA-B*57:01 | 163 | 171 | 9 |  | 0.037145 | 3.4 |  |
| HLA-A*24:02 | 163 | 171 | 9 |  | 0.029872 | 1.8 |  |
| HLA-A*23:01 | 163 | 171 | 9 |  | 0.029751 | 1.7 |  |
| HLA-B*58:01 | 163 | 171 | 9 |  | 0.022606 | 2.8 |  |
| HLA-B*15:01 | 163 | 171 | 9 |  | 0.020901 | 3.8 |  |
| HLA-A*32:01 | 163 | 171 | 9 |  | 0.012841 | 3 |  |
| HLA-A*01:01 | 163 | 171 | 9 |  | 0.00976 | 3.8 |  |
| HLA-B*44:02 | 163 | 171 | 9 |  | 0.009344 | 2.7 |  |
| HLA-B*44:03 | 163 | 171 | 9 |  | 0.006905 | 3.4 |  |
| HLA-A*30:02 | 163 | 171 | 9 |  | 0.004862 | 9.8 |  |
| HLA-A*68:02 | 163 | 171 | 9 |  | 0.004308 | 7.7 |  |
| HLA-B*40:01 | 163 | 171 | 9 |  | 0.003766 | 4.2 |  |
| HLA-A*02:06 | 163 | 171 | 9 |  | 0.00351 | 12 |  |
| HLA-A*68:01 | 163 | 171 | 9 |  | 0.003293 | 11 |  |
| HLA-A*33:01 | 163 | 171 | 9 |  | 0.002368 | 9.2 |  |
| HLA-A*03:01 | 163 | 171 | 9 |  | 0.001059 | 12 |  |
| HLA-A*02:01 | 163 | 171 | 9 |  | 0.001012 | 14 |  |
| HLA-A*30:01 | 163 | 171 | 9 |  | 0.000811 | 29 |  |
| HLA-A*31:01 | 163 | 171 | 9 |  | 0.000594 | 19 |  |
| HLA-A*11:01 | 163 | 171 | 9 |  | 0.000459 | 13 |  |
| HLA-A*02:03 | 163 | 171 | 9 |  | 0.000242 | 27 |  |
| HLA-B*40:01 | 17 | 25 | 9 | SENGHIEPL | 0.980577 | 0.01 | 94.37% |
| HLA-B*44:03 | 17 | 25 | 9 |  | 0.611471 | 0.18 |  |
| HLA-B*44:02 | 17 | 25 | 9 |  | 0.57489 | 0.15 |  |
| HLA-A*02:06 | 17 | 25 | 9 |  | 0.061436 | 2.4 |  |
| HLA-B*08:01 | 17 | 25 | 9 |  | 0.053407 | 2 |  |
| HLA-B*15:01 | 17 | 25 | 9 |  | 0.050413 | 2.5 |  |
| HLA-A*26:01 | 17 | 25 | 9 |  | 0.020296 | 2.2 |  |
| HLA-A*02:03 | 17 | 25 | 9 |  | 0.0154 | 4.6 |  |
| HLA-B*07:02 | 17 | 25 | 9 |  | 0.015286 | 3.1 |  |
| HLA-B*35:01 | 17 | 25 | 9 |  | 0.01126 | 3.5 |  |
| HLA-A*68:02 | 17 | 25 | 9 |  | 0.010918 | 4.8 |  |
| HLA-A*30:02 | 17 | 25 | 9 |  | 0.007299 | 7.6 |  |
| HLA-B*51:01 | 17 | 25 | 9 |  | 0.00667 | 7.4 |  |
| HLA-A*02:01 | 17 | 25 | 9 |  | 0.006225 | 6.2 |  |
| HLA-A*32:01 | 17 | 25 | 9 |  | 0.004185 | 5.4 |  |
| HLA-A*30:01 | 17 | 25 | 9 |  | 0.003953 | 14 |  |
| HLA-B*53:01 | 17 | 25 | 9 |  | 0.003472 | 5.4 |  |
| HLA-A*68:01 | 17 | 25 | 9 |  | 0.002153 | 12 |  |
| HLA-A*01:01 | 17 | 25 | 9 |  | 0.002125 | 9.3 |  |
| HLA-A*23:01 | 17 | 25 | 9 |  | 0.001405 | 7.2 |  |
| HLA-A*24:02 | 17 | 25 | 9 |  | 0.001238 | 7.4 |  |
| HLA-A*33:01 | 17 | 25 | 9 |  | 0.000999 | 14 |  |
| HLA-A*31:01 | 17 | 25 | 9 |  | 0.000811 | 17 |  |
| HLA-B*57:01 | 17 | 25 | 9 |  | 0.000536 | 25 |  |
| HLA-A*11:01 | 17 | 25 | 9 |  | 0.000527 | 12 |  |
| HLA-B*58:01 | 17 | 25 | 9 |  | 0.000511 | 19 |  |
| HLA-A*03:01 | 17 | 25 | 9 |  | 0.000435 | 16 |  |
| HLA-A*11:01 | 90 | 98 | 9 | GSLPIGLAK | 0.956349 | 0.01 | 99.73% |
| HLA-A*03:01 | 90 | 98 | 9 |  | 0.830849 | 0.06 |  |
| HLA-A*30:01 | 90 | 98 | 9 |  | 0.625632 | 0.06 |  |
| HLA-A*31:01 | 90 | 98 | 9 |  | 0.289765 | 0.8 |  |
| HLA-A*68:01 | 90 | 98 | 9 |  | 0.259434 | 1.4 |  |
| HLA-A*30:02 | 90 | 98 | 9 |  | 0.03945 | 2.6 |  |
| HLA-B*57:01 | 90 | 98 | 9 |  | 0.019355 | 4.6 |  |
| HLA-A*32:01 | 90 | 98 | 9 |  | 0.017072 | 2.6 |  |
| HLA-B*58:01 | 90 | 98 | 9 |  | 0.015653 | 3.4 |  |
| HLA-A*01:01 | 90 | 98 | 9 |  | 0.012472 | 3.4 |  |
| HLA-A*33:01 | 90 | 98 | 9 |  | 0.009834 | 5.1 |  |
| HLA-A*02:06 | 90 | 98 | 9 |  | 0.004015 | 11 |  |
| HLA-B*15:01 | 90 | 98 | 9 |  | 0.001976 | 11 |  |
| HLA-A*26:01 | 90 | 98 | 9 |  | 0.0015 | 8.9 |  |
| HLA-A*02:01 | 90 | 98 | 9 |  | 0.000833 | 15 |  |
| HLA-A*68:02 | 90 | 98 | 9 |  | 0.000819 | 17 |  |
| HLA-B*07:02 | 90 | 98 | 9 |  | 0.000603 | 15 |  |
| HLA-B*44:02 | 90 | 98 | 9 |  | 0.000495 | 11 |  |
| HLA-B*44:03 | 90 | 98 | 9 |  | 0.000409 | 12 |  |
| HLA-B*35:01 | 90 | 98 | 9 |  | 0.000408 | 15 |  |
| HLA-B*40:01 | 90 | 98 | 9 |  | 0.000372 | 12 |  |
| HLA-B*51:01 | 90 | 98 | 9 |  | 0.000326 | 28 |  |
| HLA-A*02:03 | 90 | 98 | 9 |  | 0.000297 | 25 |  |
| HLA-B*08:01 | 90 | 98 | 9 |  | 0.000168 | 40 |  |
| HLA-B*53:01 | 90 | 98 | 9 |  | 0.000142 | 20 |  |
| HLA-A*23:01 | 90 | 98 | 9 |  | 0.000125 | 19 |  |
| HLA-A*24:02 | 90 | 98 | 9 |  | 0.000116 | 19 |  |
| HLA-B*51:01 | 110 | 118 | 9 | IPISLTQQI | 0.981148 | 0.01 | 96.08% |
| HLA-B*53:01 | 110 | 118 | 9 |  | 0.831068 | 0.03 |  |
| HLA-B*35:01 | 110 | 118 | 9 |  | 0.587028 | 0.17 |  |
| HLA-B*07:02 | 110 | 118 | 9 |  | 0.573646 | 0.19 |  |
| HLA-B*08:01 | 110 | 118 | 9 |  | 0.106973 | 1.1 |  |
| HLA-A*68:02 | 110 | 118 | 9 |  | 0.069863 | 1.6 |  |
| HLA-B*57:01 | 110 | 118 | 9 |  | 0.014484 | 5.3 |  |
| HLA-A*02:06 | 110 | 118 | 9 |  | 0.013093 | 5.9 |  |
| HLA-A*26:01 | 110 | 118 | 9 |  | 0.011415 | 3 |  |
| HLA-B*58:01 | 110 | 118 | 9 |  | 0.010324 | 4.2 |  |
| HLA-A*24:02 | 110 | 118 | 9 |  | 0.007211 | 3.4 |  |
| HLA-A*23:01 | 110 | 118 | 9 |  | 0.006747 | 3.6 |  |
| HLA-A*02:01 | 110 | 118 | 9 |  | 0.00468 | 7.1 |  |
| HLA-A*32:01 | 110 | 118 | 9 |  | 0.003823 | 5.7 |  |
| HLA-A*30:01 | 110 | 118 | 9 |  | 0.00326 | 16 |  |
| HLA-B*44:02 | 110 | 118 | 9 |  | 0.002847 | 4.9 |  |
| HLA-A*02:03 | 110 | 118 | 9 |  | 0.002613 | 11 |  |
| HLA-B*44:03 | 110 | 118 | 9 |  | 0.002579 | 5.3 |  |
| HLA-A*01:01 | 110 | 118 | 9 |  | 0.002206 | 9.1 |  |
| HLA-B*40:01 | 110 | 118 | 9 |  | 0.001927 | 5.6 |  |
| HLA-A*30:02 | 110 | 118 | 9 |  | 0.001766 | 18 |  |
| HLA-A*68:01 | 110 | 118 | 9 |  | 0.001327 | 14 |  |
| HLA-B*15:01 | 110 | 118 | 9 |  | 0.001238 | 13 |  |
| HLA-A*33:01 | 110 | 118 | 9 |  | 0.000953 | 14 |  |
| HLA-A*31:01 | 110 | 118 | 9 |  | 0.000199 | 28 |  |
| HLA-A*03:01 | 110 | 118 | 9 |  | 0.000196 | 22 |  |
| HLA-A*11:01 | 110 | 118 | 9 |  | 0.000103 | 21 |  |
| HLA-B*57:01 | 599 | 607 | 9 | ISISGVPRY | 0.849904 | 0.16 | 100% |
| HLA-A*30:02 | 599 | 607 | 9 |  | 0.827945 | 0.02 |  |
| HLA-B*58:01 | 599 | 607 | 9 |  | 0.825446 | 0.1 |  |
| HLA-B*15:01 | 599 | 607 | 9 |  | 0.64373 | 0.18 |  |
| HLA-B*35:01 | 599 | 607 | 9 |  | 0.485491 | 0.24 |  |
| HLA-A*01:01 | 599 | 607 | 9 |  | 0.463151 | 0.21 |  |
| HLA-A*26:01 | 599 | 607 | 9 |  | 0.34198 | 0.21 |  |
| HLA-A*11:01 | 599 | 607 | 9 |  | 0.235991 | 0.78 |  |
| HLA-A*32:01 | 599 | 607 | 9 |  | 0.183192 | 0.42 |  |
| HLA-B*53:01 | 599 | 607 | 9 |  | 0.123562 | 0.64 |  |
| HLA-A*30:01 | 599 | 607 | 9 |  | 0.108535 | 1.5 |  |
| HLA-A*03:01 | 599 | 607 | 9 |  | 0.097252 | 1.6 |  |
| HLA-A*68:01 | 599 | 607 | 9 |  | 0.08196 | 2.8 |  |
| HLA-B*51:01 | 599 | 607 | 9 |  | 0.02203 | 3.8 |  |
| HLA-A*31:01 | 599 | 607 | 9 |  | 0.021362 | 4.5 |  |
| HLA-A*02:06 | 599 | 607 | 9 |  | 0.011959 | 6.3 |  |
| HLA-B*44:03 | 599 | 607 | 9 |  | 0.005786 | 3.7 |  |
| HLA-A*23:01 | 599 | 607 | 9 |  | 0.004638 | 4.3 |  |
| HLA-B*44:02 | 599 | 607 | 9 |  | 0.004544 | 3.9 |  |
| HLA-A*24:02 | 599 | 607 | 9 |  | 0.004411 | 4.3 |  |
| HLA-A*33:01 | 599 | 607 | 9 |  | 0.003426 | 7.8 |  |
| HLA-A*68:02 | 599 | 607 | 9 |  | 0.00314 | 9 |  |
| HLA-A*02:01 | 599 | 607 | 9 |  | 0.00177 | 11 |  |
| HLA-A*02:03 | 599 | 607 | 9 |  | 0.001146 | 15 |  |
| HLA-B*40:01 | 599 | 607 | 9 |  | 0.001072 | 7.3 |  |
| HLA-B*08:01 | 599 | 607 | 9 |  | 0.000792 | 22 |  |
| HLA-B*07:02 | 599 | 607 | 9 |  | 0.000494 | 16 |  |

Table S3: Most potential non-allergen, nontoxic, 05 T-cell epitopes with interacting MHC-I alleles, epitope conservancy score

Table S4: Most potential non-allergen, nontoxic, 11 T-cell epitopes with interacting MHC-I alleles, epitope conservancy score

| Allele | Start | End | Length | Peptide | Percentile rank | Adjusted rank | Conservancy |
| --- | --- | --- | --- | --- | --- | --- | --- |
| HLA-DRB5*01:01 | 57 | 71 | 15 | VFLLGFFEMERIKDK | 17 | 17 | 80.70% |
| HLA-DRB1*15:01 | 57 | 71 | 15 |  | 20 | 20 |  |
| HLA-DRB1*03:01 | 57 | 71 | 15 |  | 26 | 26 |  |
| HLA-DRB4*01:01 | 57 | 71 | 15 |  | 32 | 32 |  |
| HLA-DRB3*01:01 | 57 | 71 | 15 |  | 39 | 39 |  |
| HLA-DRB3*02:02 | 57 | 71 | 15 |  | 44 | 44 |  |
| HLA-DRB1*07:01 | 57 | 71 | 15 |  | 55 | 55 |  |
| HLA-DRB1*15:01 | 333 | 347 | 15 | GEFRYYPNIIAKGVG | 0.15 | 0.15 | 99.73% |
| HLA-DRB3*02:02 | 333 | 347 | 15 |  | 0.57 | 0.57 |  |
| HLA-DRB1*07:01 | 333 | 347 | 15 |  | 19 | 19 |  |
| HLA-DRB5*01:01 | 333 | 347 | 15 |  | 19 | 19 |  |
| HLA-DRB3*01:01 | 333 | 347 | 15 |  | 23 | 23 |  |
| HLA-DRB4*01:01 | 333 | 347 | 15 |  | 41 | 41 |  |
| HLA-DRB1*03:01 | 333 | 347 | 15 |  | 59 | 59 |  |
| HLA-DRB1*15:01 | 329 | 343 | 15 | PSLPGEFRYYPNIIA | 0.15 | 0.15 | 100.00% |
| HLA-DRB3*02:02 | 329 | 343 | 15 |  | 12 | 12 |  |
| HLA-DRB1*07:01 | 329 | 343 | 15 |  | 19 | 19 |  |
| HLA-DRB5*01:01 | 329 | 343 | 15 |  | 22 | 22 |  |
| HLA-DRB3*01:01 | 329 | 343 | 15 |  | 23 | 23 |  |
| HLA-DRB4*01:01 | 329 | 343 | 15 |  | 53 | 53 |  |
| HLA-DRB1*03:01 | 329 | 343 | 15 |  | 65 | 65 |  |
| HLA-DRB1*15:01 | 334 | 348 | 15 | EFRYYPNIIAKGVGK | 0.17 | 0.17 | 99.46% |
| HLA-DRB3*02:02 | 334 | 348 | 15 |  | 0.64 | 0.64 |  |
| HLA-DRB5*01:01 | 334 | 348 | 15 |  | 17 | 17 |  |
| HLA-DRB1*07:01 | 334 | 348 | 15 |  | 19 | 19 |  |
| HLA-DRB3*01:01 | 334 | 348 | 15 |  | 23 | 23 |  |
| HLA-DRB4*01:01 | 334 | 348 | 15 |  | 41 | 41 |  |
| HLA-DRB1*03:01 | 334 | 348 | 15 |  | 61 | 61 |  |
| HLA-DRB1*07:01 | 33 | 47 | 15 | TYILWTITLVLLSIV | 7.7 | 7.7 | 91.18% |
| HLA-DRB1*15:01 | 33 | 47 | 15 |  | 18 | 18 |  |
| HLA-DRB4*01:01 | 33 | 47 | 15 |  | 20 | 20 |  |
| HLA-DRB3*01:01 | 33 | 47 | 15 |  | 28 | 28 |  |
| HLA-DRB1*03:01 | 33 | 47 | 15 |  | 36 | 36 |  |
| HLA-DRB3*02:02 | 33 | 47 | 15 |  | 42 | 42 |  |
| HLA-DRB5*01:01 | 33 | 47 | 15 |  | 43 | 43 |  |
| HLA-DRB3*02:02 | 45 | 59 | 15 | SIVFIIVLTNSIKSE | 1.6 | 1.6 | 70.59% |
| HLA-DRB5*01:01 | 45 | 59 | 15 |  | 2 | 2 |  |
| HLA-DRB1*15:01 | 45 | 59 | 15 |  | 3.8 | 3.8 |  |
| HLA-DRB1*07:01 | 45 | 59 | 15 |  | 4.3 | 4.3 |  |
| HLA-DRB4*01:01 | 45 | 59 | 15 |  | 4.7 | 4.7 |  |
| HLA-DRB1*03:01 | 45 | 59 | 15 |  | 8.7 | 8.7 |  |
| HLA-DRB3*01:01 | 45 | 59 | 15 |  | 17 | 17 |  |
| HLA-DRB1*07:01 | 34 | 48 | 15 | YILWTITLVLLSIVF | 7.7 | 7.7 | 91.18% |
| HLA-DRB1*15:01 | 34 | 48 | 15 |  | 18 | 18 |  |
| HLA-DRB4*01:01 | 34 | 48 | 15 |  | 20 | 20 |  |
| HLA-DRB1*03:01 | 34 | 48 | 15 |  | 37 | 37 |  |
| HLA-DRB3*01:01 | 34 | 48 | 15 |  | 38 | 38 |  |
| HLA-DRB5*01:01 | 34 | 48 | 15 |  | 39 | 39 |  |
| HLA-DRB3*02:02 | 34 | 48 | 15 |  | 50 | 50 |  |
| HLA-DRB1*07:01 | 201 | 215 | 15 | GEIELLKRLTTISIS | 1.8 | 1.8 | 100% |
| HLA-DRB4*01:01 | 201 | 215 | 15 |  | 2.4 | 2.4 |  |
| HLA-DRB1*15:01 | 201 | 215 | 15 |  | 6 | 6 |  |
| HLA-DRB1*03:01 | 201 | 215 | 15 |  | 15 | 15 |  |
| HLA-DRB3*02:02 | 201 | 215 | 15 |  | 16 | 16 |  |
| HLA-DRB5*01:01 | 201 | 215 | 15 |  | 29 | 29 |  |
| HLA-DRB3*01:01 | 201 | 215 | 15 |  | 33 | 33 |  |
| HLA-DRB1*07:01 | 61 | 75 | 15 | YQSFIGIKFNKFIEP | 11 | 11 | 100% |
| HLA-DRB1*15:01 | 61 | 75 | 15 |  | 11 | 11 |  |
| HLA-DRB5*01:01 | 61 | 75 | 15 |  | 11 | 11 |  |
| HLA-DRB3*02:02 | 61 | 75 | 15 |  | 13 | 13 |  |
| HLA-DRB4*01:01 | 61 | 75 | 15 |  | 16 | 16 |  |
| HLA-DRB3*01:01 | 61 | 75 | 15 |  | 22 | 22 |  |
| HLA-DRB1*03:01 | 61 | 75 | 15 |  | 58 | 58 |  |
| HLA-DRB4*01:01 | 64 | 78 | 15 | FIGIKFNKFIEPQLD | 16 | 16 | 100% |
| HLA-DRB5*01:01 | 64 | 78 | 15 |  | 16 | 16 |  |
| HLA-DRB1*15:01 | 64 | 78 | 15 |  | 20 | 20 |  |
| HLA-DRB3*02:02 | 64 | 78 | 15 |  | 20 | 20 |  |
| HLA-DRB1*07:01 | 64 | 78 | 15 |  | 23 | 23 |  |
| HLA-DRB3*01:01 | 64 | 78 | 15 |  | 46 | 46 |  |
| HLA-DRB1*03:01 | 64 | 78 | 15 |  | 62 | 62 |  |
| HLA-DRB1*15:01 | 63 | 77 | 15 | SFIGIKFNKFIEPQL | 15 | 15 | 100% |
| HLA-DRB3*02:02 | 63 | 77 | 15 |  | 15 | 15 |  |
| HLA-DRB4*01:01 | 63 | 77 | 15 |  | 15 | 15 |  |
| HLA-DRB5*01:01 | 63 | 77 | 15 |  | 15 | 15 |  |
| HLA-DRB1*07:01 | 63 | 77 | 15 |  | 22 | 22 |  |
| HLA-DRB3*01:01 | 63 | 77 | 15 |  | 37 | 37 |  |
| HLA-DRB1*03:01 | 63 | 77 | 15 |  | 57 | 57 |  |

Table S5: Predicted Linear Epitope(s): ElliPro

| No. | Chain | Start | End | Peptide | Number of residues | Score |
| --- | --- | --- | --- | --- | --- | --- |
| 1 | A | 132 | 180 | PGPGGEFRYYPNIIAKGVGGPGPGPSLPGEFRYYPNIIAGPGPGEFRYY | 49 | 0.799 |
| 2 | A | 441 | 478 | WGHSCKKRERHGGQWPPVTLPDHAHKKSLKEKEIKQEG | 38 | 0.778 |
| 3 | A | 365 | 419 | KKVDERSDYASSGIKKEHPINENAICNTTGCPGKTQRDCNQASHSPWFSDKKDYS | 55 | 0.747 |
| 4 | A | 60 | 82 | IKFAAYSENGHIEPLAAYGSLPI | 23 | 0.734 |
| 5 | A | 285 | 325 | NKFIEPGPGPGFIGIKFNKFIEPQLDGPGPGSFIGIKFNKF | 41 | 0.707 |
| 6 | A | 89 | 113 | YIPISLTQQIAAYISISGVPRYGPG | 25 | 0.592 |
| 7 | A | 267 | 277 | ISISGPGPGYQ | 11 | 0.589 |
| 8 | A | 186 | 195 | KGVGKGPGPG | 10 | 0.556 |
| 9 | A | 229 | 235 | SEGPGPG | 7 | 0.52 |

Table S6: Predicted Discontinuous Epitope(s): ElliPro

| No. | Residues | Number of residues | Score |
| --- | --- | --- | --- |
| 19 | A:A88, A:Y89, A:I90, A:P91, A:I92, A:S93, A:L94, A:T95, A:Q96 | 9 | 0.521 |
| 9 | A:E72, A:P73, A:L74, A:A75, A:A76, A:Y77, A:G78, A:S79, A:L80, A:P81, A:I82 | 11 | 0.717 |
| 11 | A:E289, A:P290, A:G291, A:P292, A:G293, A:P294, A:G295, A:F296, A:I297, A:G298, A:I299, A:K300, A:F301 | 13 | 0.688 |
| 5 | A:E306, A:P307, A:Q308, A:L309, A:D310, A:G311, A:P312, A:G313, A:P314, A:G315, A:S316, A:F317, A:I318, A:G319, A:I320, A:K321, A:F322 | 17 | 0.818 |
| 20 | A:F284, A:F287, A:I288 | 3 | 0.5 |
| 16 | A:F412, A:S413, A:D414, A:K415, A:K416 | 5 | 0.574 |
| 13 | A:G442, A:H443, A:S444, A:C445, A:K446, A:K447 | 6 | 0.606 |
| 4 | A:H382, A:P383, A:I384, A:N385, A:E386, A:N387, A:A388, A:I389, A:C390, A:N391, A:T392, A:T393, A:G394, A:C395, A:P396, A:G397, A:K398, A:T399, A:Q400, A:R401, A:D402, A:C403, A:N404, A:Q405, A:A406, A:S407, A:H408, A:S409, A:P410, A:W411 | 30 | 0.825 |
| 15 | A:I267, A:S268, A:I269, A:S270, A:G271, A:P272, A:G273, A:P274, A:G275, A:Y276, A:Q277, A:S278 | 12 | 0.576 |
| 7 | A:K61, A:F62, A:A63, A:A64, A:Y65, A:S66, A:E67, A:N68, A:G69, A:H70, A:I71 | 11 | 0.781 |
| 17 | A:K186, A:G187, A:V188, A:G189, A:K190, A:G191, A:P192, A:G193, A:P194, A:G195 | 10 | 0.556 |
| 10 | A:K366, A:V367, A:D368, A:E369, A:R370, A:S371, A:D372, A:Y373, A:A374, A:S375, A:S376, A:G377, A:I378, A:K379 | 14 | 0.696 |
| 2 | A:K467, A:S468, A:L469, A:K470, A:E471, A:K472 | 6 | 0.865 |
| 1 | A:K475, A:Q476, A:E477, A:G478 | 4 | 0.99 |
| 3 | A:P132, A:G133, A:P134, A:G135, A:G136, A:E137, A:F138, A:R139, A:Y140, A:Y141, A:P142, A:N143, A:I144, A:I145, A:A146, A:K147, A:G148, A:V149, A:G150, A:G151, A:P152, A:G153, A:P154, A:G155, A:P156, A:S157, A:L158, A:P159, A:G160, A:E161, A:F162, A:R163 | 32 | 0.841 |
| 12 | A:Q97, A:I98, A:A99, A:A100, A:Y101, A:I102, A:S103, A:I104, A:S105, A:G106, A:V107, A:P108, A:R109 | 13 | 0.628 |
| 8 | A:R450, A:H451, A:G452, A:G453, A:Q454, A:W455, A:P456, A:P457, A:V458, A:T459, A:L460, A:P461, A:D462, A:H463, A:A464, A:H465, A:K466 | 17 | 0.769 |
| 18 | A:S229, A:E230, A:G231, A:P232, A:G233, A:P234, A:G235 | 7 | 0.522 |
| 14 | A:Y110, A:G111, A:P112, A:G113 | 4 | 0.603 |
| 6 | A:Y164, A:Y165, A:P166, A:N167, A:I168, A:I169, A:A170, A:G171, A:P172, A:G173, A:P174, A:G175, A:E176 | 13 | 0.798 |

| population/area | Class I | | | Class II | | | Class combined | | |
| --- | --- | --- | --- | --- | --- | --- | --- | --- | --- |
|  | **coverage^a^** | **average_hit^b^** | **pc90^c^** | **coverage^a^** | **average_hit^b^** | **pc90^c^** | **coverage^a^** | **average**  **hit^b^** | **pc90^c^** |
| [World](http://tools.iedb.org/population/result/#World) | 98.55% | 11.49 | 7.57 | 49.02% | 5.99 | 2.16 | 99.26% | 17.49 | 10.04 |
|  |  |  |  |  |  |  |  |  |  |
| Average | **98.55** | **11.49** | **7.57** | **49.02** | **5.99** | **2.16** | **99.26** | **17.49** | **10.04** |
| Standard deviation | **0.0** | **0.0** | **0.0** | **0.0** | **0.0** | **0.0** | **0.0** | **0.0** | **0.0** |

Table S7: Population Coverage Calculation Result

^a^ projected population coverage

^b^ average number of epitope hits / HLA combinations recognized by the population

^c^ minimum number of epitope hits / HLA combinations recognized by 90% of the population


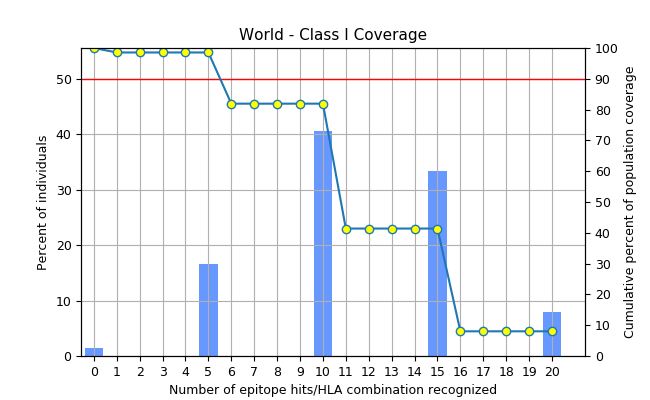

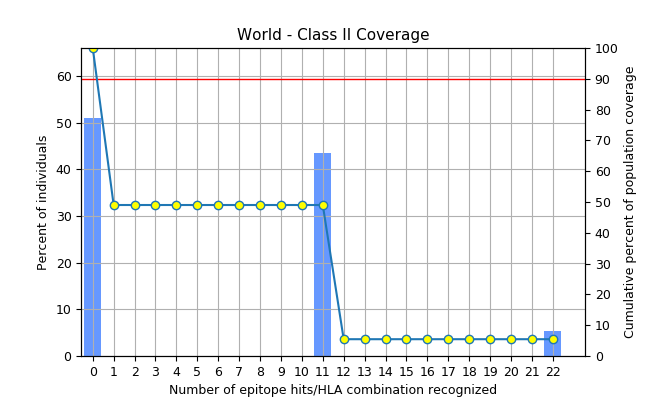


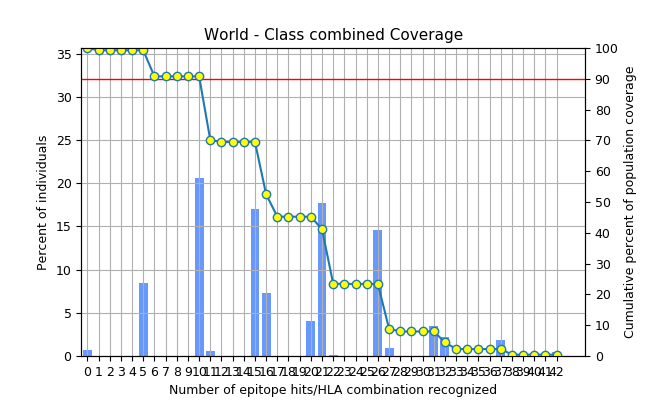


Figure S1: Human population coverage analysis

Alpha helix (Hh) : 92 is 19.25%

3_10_  helix (Gg) : 0 is 0.00%

Pi helix (Ii) : 0 is 0.00%

Beta bridge (Bb) : 0 is 0.00%

Extended strand (Ee) : 120 is 25.10%

Beta turn (Tt) : 35 is 7.32%

Bend region (Ss) : 0 is 0.00%

Random coil (Cc) : 231 is 48.33%

Ambiguous states (?) : 0 is 0.00%


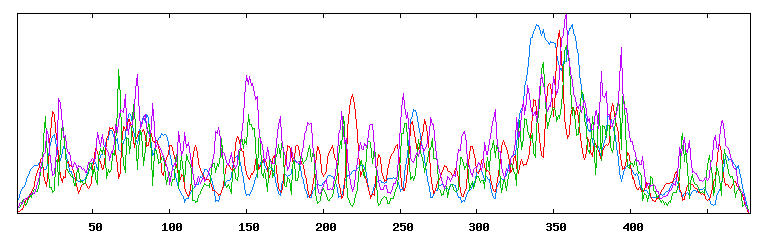
 Other states : 0 is 0.00%

Figure S2: Secondary Structure Prediction by SOPMA.


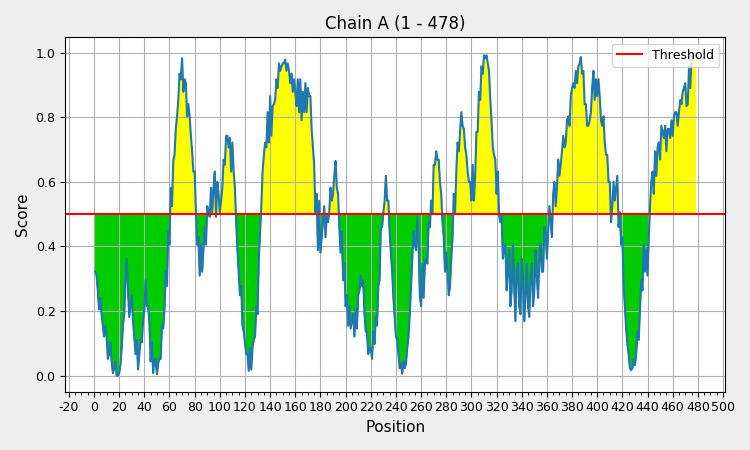


Figure S3: Conformational B-cell epitopes.


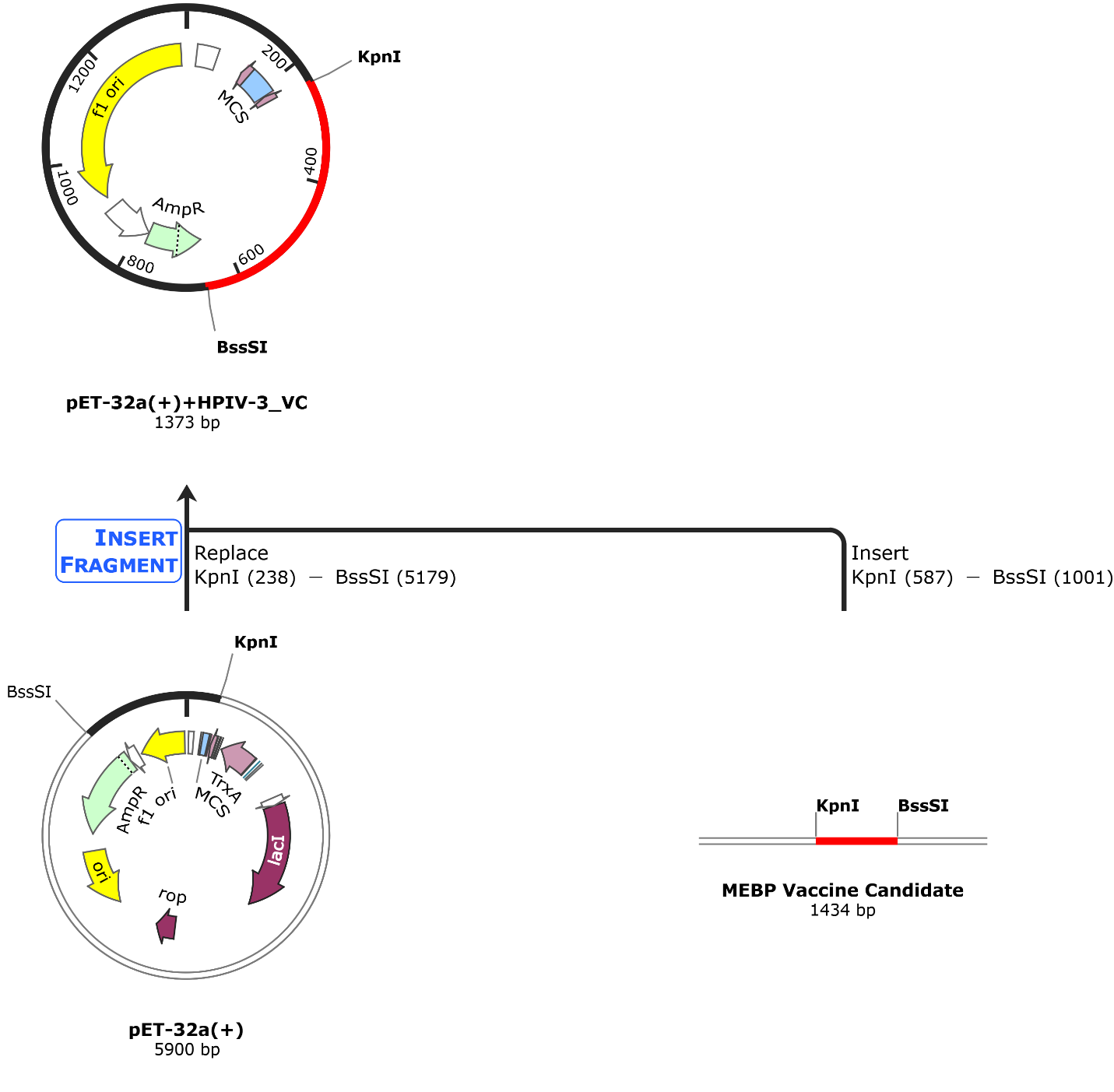


Figure S4: Cloning in pET-32 (+) vector after restriction site addition and in silico PCR amplification of the vaccine construct.
