# Supplementary material for: Immunoinformatics-aided rational design of multiepitope-based peptide vaccine (MEBV) targeting human parainfluenza virus 3 (HPIV-3) stable proteins
Source: J Genet Eng Biotechnol. 2023 Dec 6;21:162. doi: 10.1186/s43141-023-00623-5 (PMC10700276; doi:10.1186/s43141-023-00623-5)
Supplement: Supplementary file 2 — Additional file 2. [file 43141_2023_623_MOESM2_ESM.pdf]

# GalaxyWEB

A web server for protein structure prediction, refinement, and related methods

Computational Biology Lab, Department of Chemistry, Seoul National University

Home Services Database Queue Help Softwares Suppl

## Predicted 3D model by Alphafold

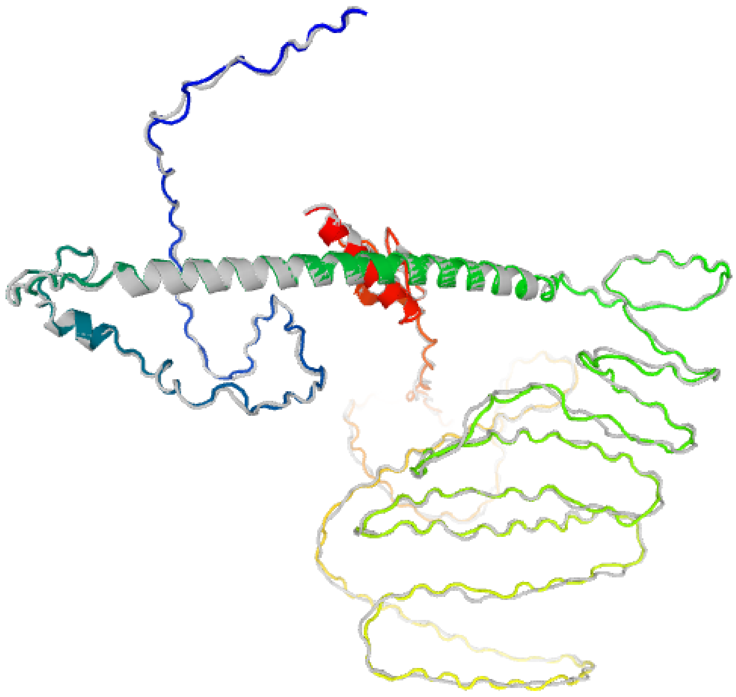

**View in PV** [Model 1] [Model 2] [Model 3] [Model 4] [Model 5] [All Models]

**Download** [Model 1] [Model 2] [Model 3] [Model 4] [Model 5] [All Models]

### Structure Information

| Model | GDT-HA | RMSD | MolProbity | Clash score | Poor rotamers | Rama favored |
|---|---|---|---|---|---|---|
| Initial | 1.0000 | 0.000 | 3.629 | 23.2 | 10.7 | 46.2 |
| MODEL 1 | 0.7892 | 0.894 | 1.201 | 2.0 | 0.0 | 96.4 |
| MODEL 2 | 0.7965 | 0.855 | 1.404 | 2.4 | 0.3 | 94.3 |
| MODEL 3 | 0.8117 | 0.828 | 1.367 | 2.4 | 0.3 | 95.0 |
| MODEL 4 | 0.7997 | 0.854 | 1.314 | 2.0 | 0.3 | 95.0 |
| MODEL 5 | 0.8023 | 0.843 | 1.415 | 2.8 | 0.0 | 95.0 |

### Download

- 5 refined models [DOWNLOAD]

Help

- **Information**
- The data will be stored in the server only for 30 days.

compbio.galaxy@gmail.com | Lab. of Computational Biology and Biomolecular Engineering
