# Supplementary material for: Immunoinformatics-aided rational design of multiepitope-based peptide vaccine (MEBV) targeting human parainfluenza virus 3 (HPIV-3) stable proteins
Source: J Genet Eng Biotechnol. 2023 Dec 6;21:162. doi: 10.1186/s43141-023-00623-5 (PMC10700276; doi:10.1186/s43141-023-00623-5)
Supplement: Supplementary file 4 — Additional file 4. [file 43141_2023_623_MOESM4_ESM.pdf]

Table of Contents

TLR3 docked with ref_Structure	2
TLR3 docked with ref	5
TLR4 docked with ref_Structure	8
TLR4 docked with ref	11
TLR8 docked with ref_Structure	14
TLR8 docked with ref	17

Dimer Classification | Queue | **Results** | Preferences | Downloads | Papers | Help | Contact
Dock | Peptide Docking

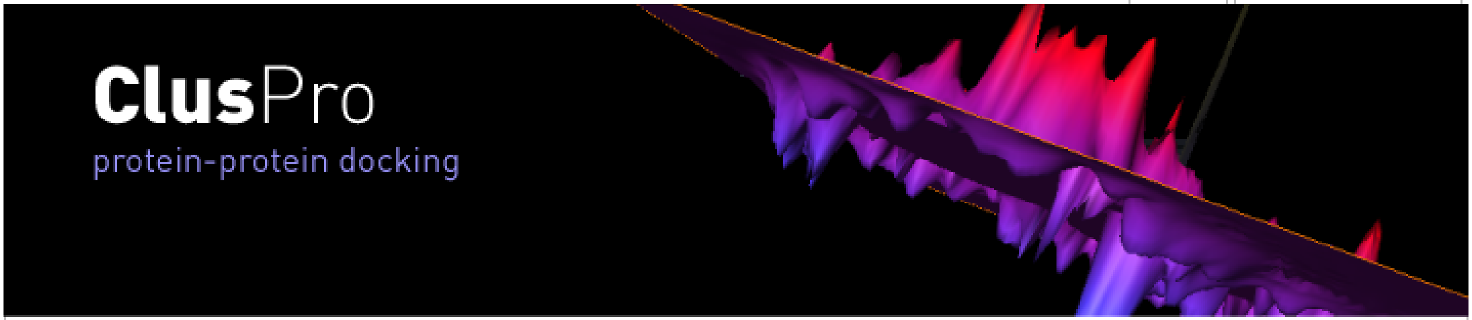

sign out

Job Details: TLR3 docked with ref

View Model Scores

Download all Models for all Coefficients

Balanced | Electrostatic-favored | Hydrophobic-favored | VdW+Elec

Display Models: 10

Download Displayed Models

If you use these models in a paper, please cite our papers

0

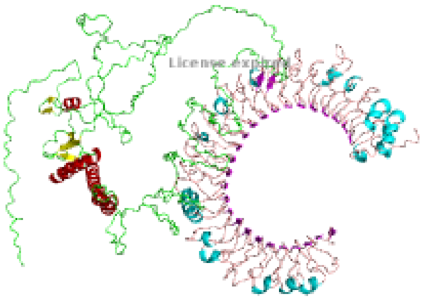

1

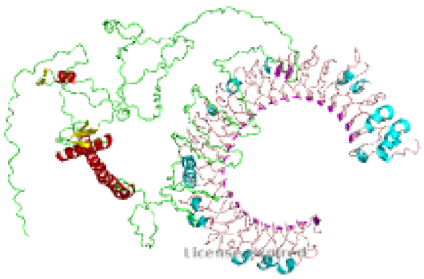

2

3

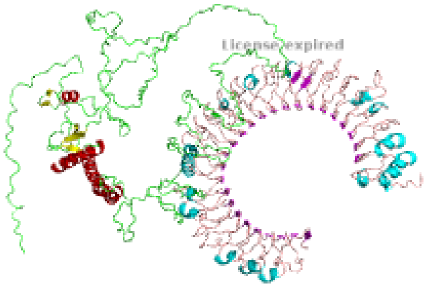

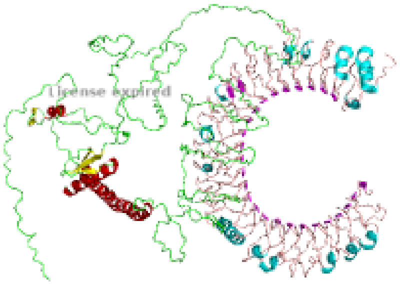

4

5

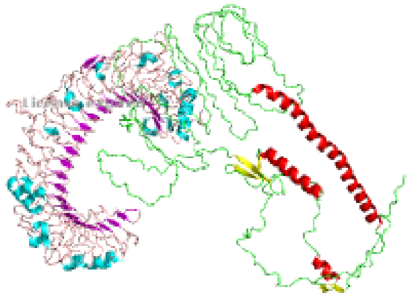

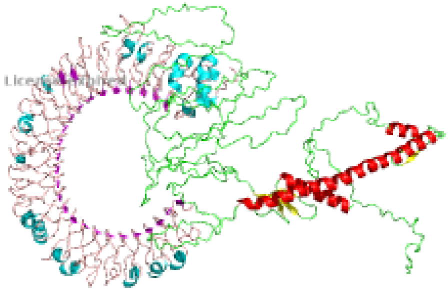

6

7

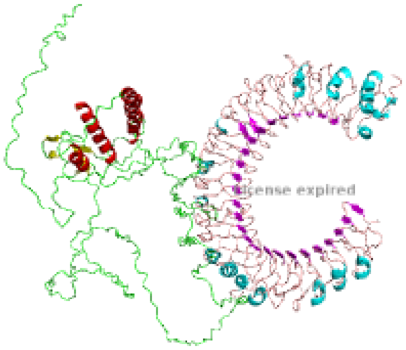

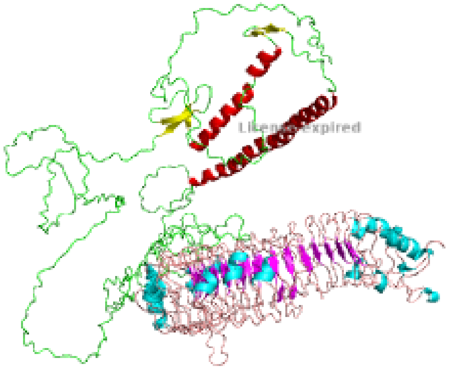

8

9

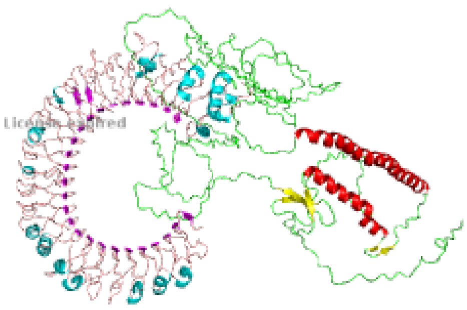

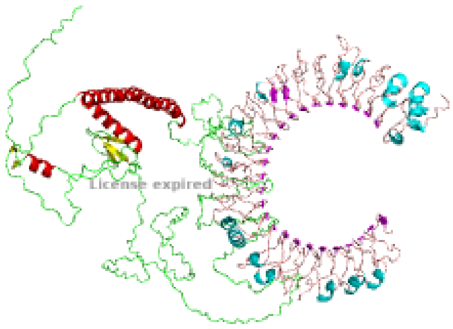

ClusPro should only be used for noncommercial purposes.
Vajda Lab and ABC Group
Boston University and Stony Brook University

ClusPro
protein-protein docking

Job Details: TLR3 docked with ref

View Models

Balanced | Electrostatic-favored | Hydrophobic-favored | VdW+Elec

Download Model Scores for this Coefficient

Coefficient Weights

See *Kozakov et. al.* in Papers for a description of these terms

$$E = 0.40E_{rep} + -0.40E_{att} + 600E_{elec} + 1.00E_{DARS}$$

Cluster Scores

We strongly encourage you to read the FAQ related to these scores before using them.

Cluster	Members	Representative	Weighted Score
**0**	44	Center	-1459.2
		Lowest Energy	-1622.1
**1**	39	Center	-1545.5
		Lowest Energy	-1754.6
**2**	34	Center	-1241.3
		Lowest Energy	-1550.9
**3**	32	Center	-1543.5
		Lowest Energy	-1734.9
**4**	32	Center	-1452.7
		Lowest Energy	-1560.1
**5**	30	Center	-1372.6
		Lowest Energy	-1488.5
**6**	27	Center	-1365.8
		Lowest Energy	-1408.5
**7**	27	Center	-1375.4

Cluster	Members	Representative	Weighted Score
		Lowest Energy	-1473.5
**8**	21	Center	-1232.0
		Lowest Energy	-1469.3
**9**	21	Center	-1341.0
		Lowest Energy	-1497.0
**10**	20	Center	-1502.2
		Lowest Energy	-1502.2
**11**	20	Center	-1226.8
		Lowest Energy	-1393.6
**12**	19	Center	-1532.5
		Lowest Energy	-1532.5
**13**	18	Center	-1270.3
		Lowest Energy	-1427.2
**14**	18	Center	-1357.0
		Lowest Energy	-1357.0
**15**	17	Center	-1319.1
		Lowest Energy	-1430.8
**16**	17	Center	-1403.8
		Lowest Energy	-1457.1
**17**	16	Center	-1254.7
		Lowest Energy	-1511.4
**18**	16	Center	-1338.3
		Lowest Energy	-1412.4
**19**	14	Center	-1589.3
		Lowest Energy	-1775.0
**20**	14	Center	-1553.1
		Lowest Energy	-1561.0
**21**	14	Center	-1506.8
		Lowest Energy	-1506.8
**22**	14	Center	-1274.7
		Lowest Energy	-1367.4
**23**	13	Center	-1237.0
		Lowest Energy	-1534.6
**24**	13	Center	-1321.7
		Lowest Energy	-1755.3
**25**	12	Center	-1273.1
		Lowest Energy	-1391.3

Cluster	Members	Representative	Weighted Score
**26**	12	Center	-1390.8
		Lowest Energy	-1472.3
**27**	11	Center	-1334.0
		Lowest Energy	-1365.0
**28**	11	Center	-1367.4
		Lowest Energy	-1425.9
**29**	11	Center	-1311.8
		Lowest Energy	-1391.8

ClusPro should only be used for noncommercial purposes.
Vajda Lab and ABC Group
Boston University and Stony Brook University

Dimer Classification | Queue | **Results** | Preferences | Downloads | Papers | Help | Contact | Dock | Peptide Docking

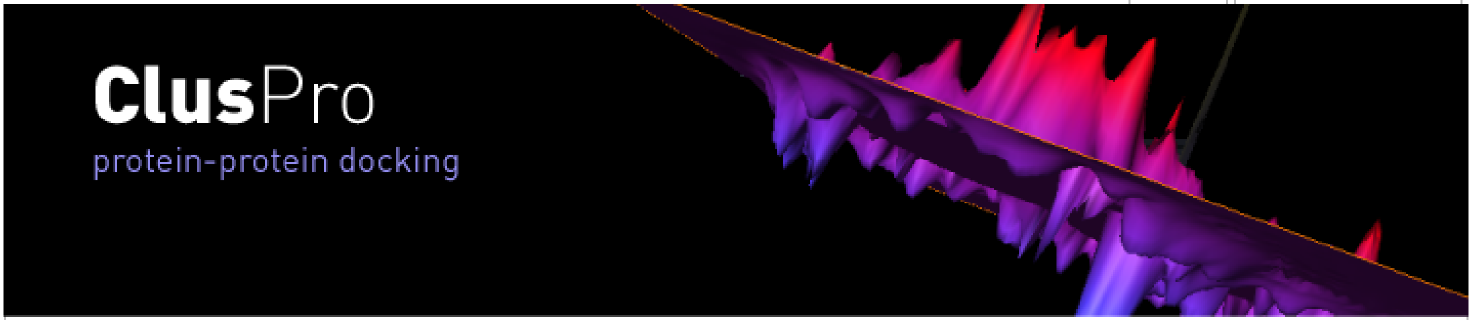

sign out

Job Details: TLR4 docked with ref

View Model Scores

Download all Models for all Coefficients

Balanced | Electrostatic-favored | Hydrophobic-favored | VdW+Elec

Display Models: 10

Download Displayed Models

If you use these models in a paper, please cite our papers

0

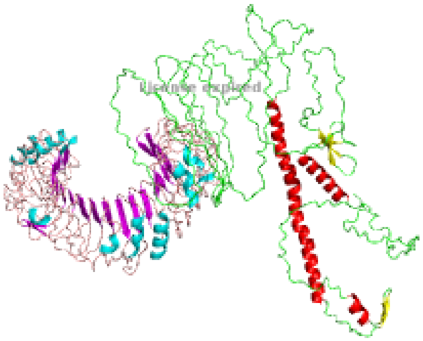

1

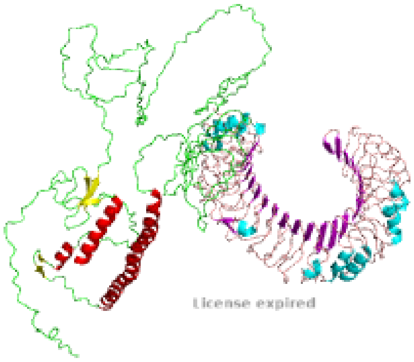

2

3

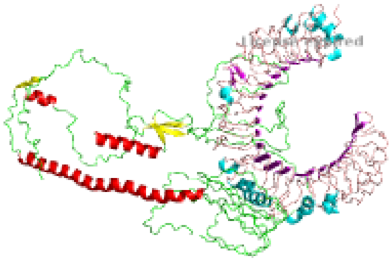

4

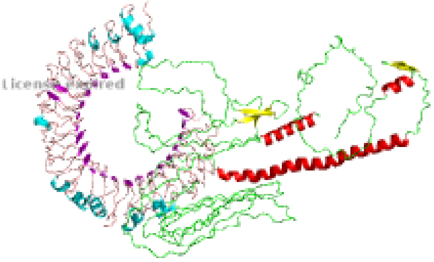

5

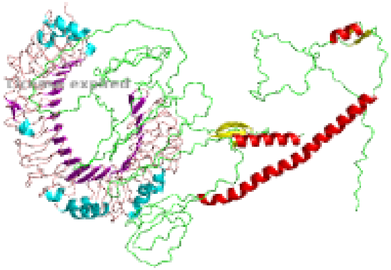

6

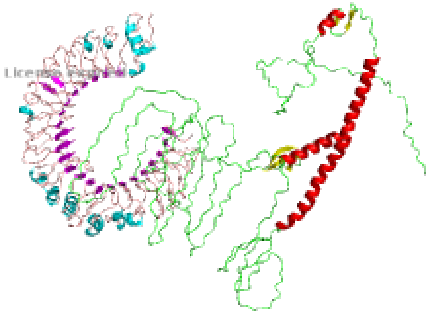

7

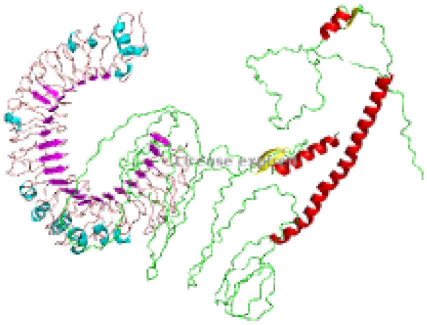

8

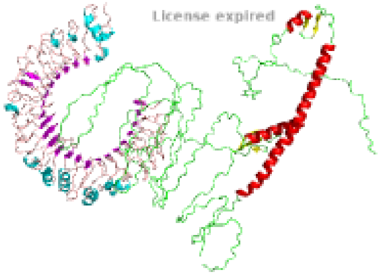

9

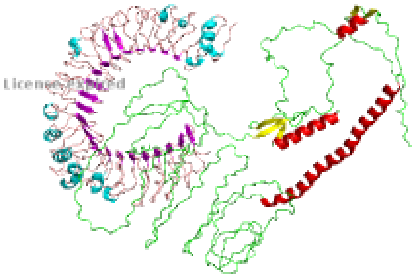

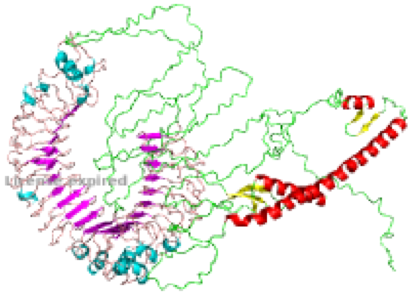

ClusPro should only be used for noncommercial purposes.
Vajda Lab and ABC Group
Boston University and Stony Brook University

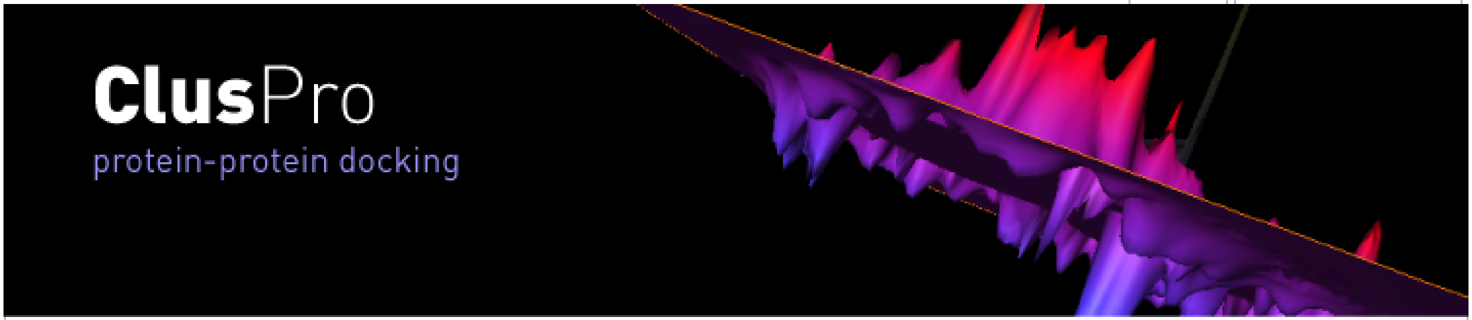

Job Details: TLR3 docked with ref

View Models

Balanced | Electrostatic-favored | Hydrophobic-favored | VdW+Elec

Download Model Scores for this Coefficient

Coefficient Weights

See *Kozakov et. al.* in Papers for a description of these terms

$$E = 0.40E_{rep} + -0.40E_{att} + 600E_{elec} + 1.00E_{DARS}$$

Cluster Scores

We strongly encourage you to read the FAQ related to these scores before using them.

Cluster	Members	Representative	Weighted Score
**0**	44	Center	-1459.2
		Lowest Energy	-1622.1
**1**	39	Center	-1545.5
		Lowest Energy	-1754.6
**2**	34	Center	-1241.3
		Lowest Energy	-1550.9
**3**	32	Center	-1543.5
		Lowest Energy	-1734.9
**4**	32	Center	-1452.7
		Lowest Energy	-1560.1
**5**	30	Center	-1372.6
		Lowest Energy	-1488.5
**6**	27	Center	-1365.8
		Lowest Energy	-1408.5
**7**	27	Center	-1375.4

Cluster	Members	Representative	Weighted Score
		Lowest Energy	-1473.5
**8**	21	Center	-1232.0
		Lowest Energy	-1469.3
**9**	21	Center	-1341.0
		Lowest Energy	-1497.0
**10**	20	Center	-1502.2
		Lowest Energy	-1502.2
**11**	20	Center	-1226.8
		Lowest Energy	-1393.6
**12**	19	Center	-1532.5
		Lowest Energy	-1532.5
**13**	18	Center	-1270.3
		Lowest Energy	-1427.2
**14**	18	Center	-1357.0
		Lowest Energy	-1357.0
**15**	17	Center	-1319.1
		Lowest Energy	-1430.8
**16**	17	Center	-1403.8
		Lowest Energy	-1457.1
**17**	16	Center	-1254.7
		Lowest Energy	-1511.4
**18**	16	Center	-1338.3
		Lowest Energy	-1412.4
**19**	14	Center	-1589.3
		Lowest Energy	-1775.0
**20**	14	Center	-1553.1
		Lowest Energy	-1561.0
**21**	14	Center	-1506.8
		Lowest Energy	-1506.8
**22**	14	Center	-1274.7
		Lowest Energy	-1367.4
**23**	13	Center	-1237.0
		Lowest Energy	-1534.6
**24**	13	Center	-1321.7
		Lowest Energy	-1755.3
**25**	12	Center	-1273.1
		Lowest Energy	-1391.3

Cluster	Members	Representative	Weighted Score
**26**	12	Center	-1390.8
		Lowest Energy	-1472.3
**27**	11	Center	-1334.0
		Lowest Energy	-1365.0
**28**	11	Center	-1367.4
		Lowest Energy	-1425.9
**29**	11	Center	-1311.8
		Lowest Energy	-1391.8

ClusPro should only be used for noncommercial purposes.
Vajda Lab and ABC Group
Boston University and Stony Brook University

Dimer Classification | Queue | **Results** | Preferences | Downloads | Papers | Help | Contact | Dock | Peptide Docking

ClusPro
protein-protein docking

sign out

Job Details: 914958

View Model Scores

Download all Models for all Coefficients

Balanced | Electrostatic-favored | Hydrophobic-favored | VdW+Elec

Display Models: 10

Download Displayed Models

If you use these models in a paper, please cite our papers

0

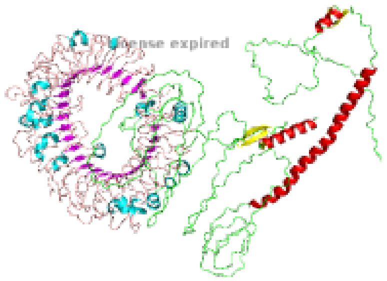

1

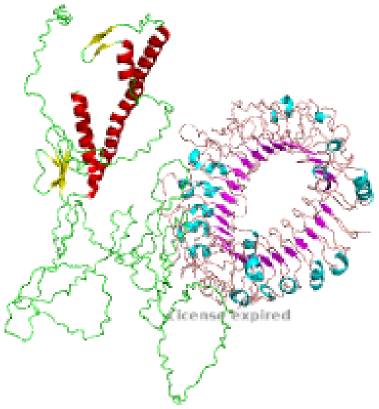

2

3

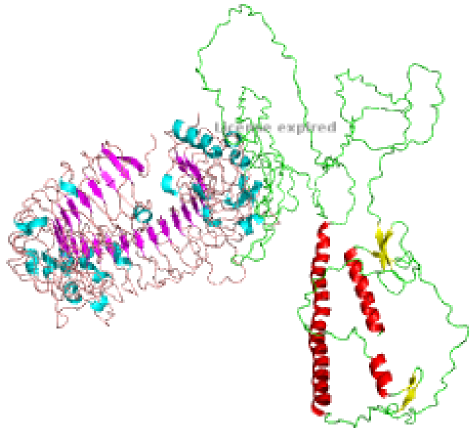
License expired

4

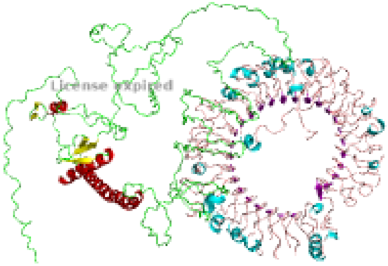
License expired

5

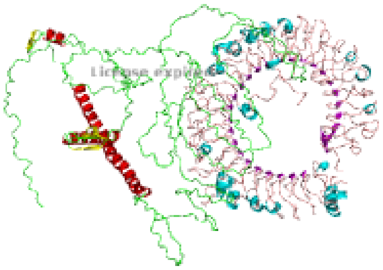
License expired

6

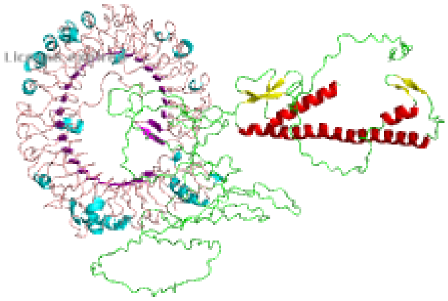

7

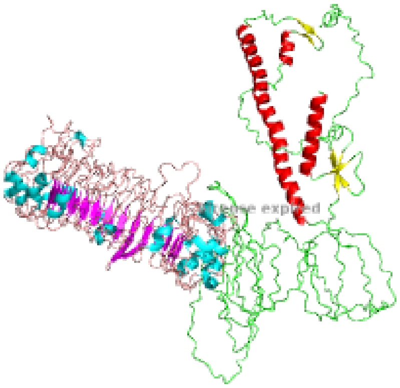
License expired

8

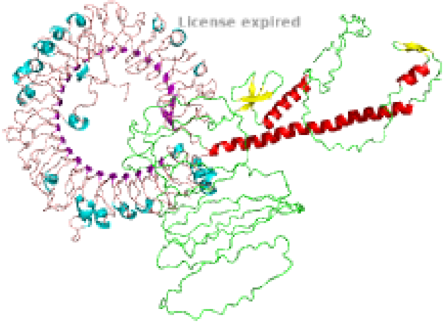
License expired

9

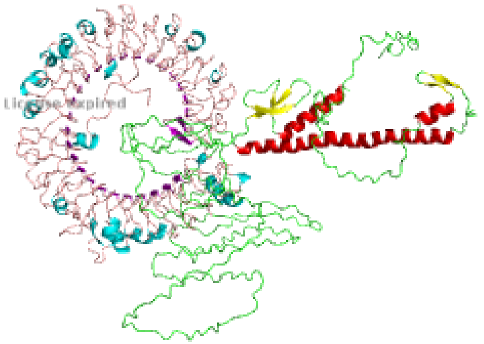

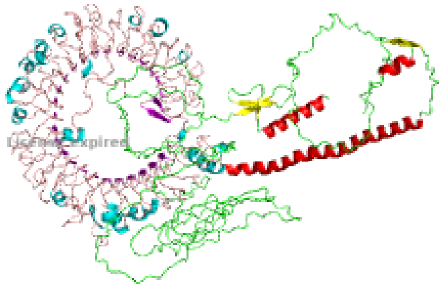

ClusPro should only be used for noncommercial purposes.
Vajda Lab and ABC Group
Boston University and Stony Brook University

Dimer Classification | Queue | **Results** | Preferences | Downloads | Papers | Help | Contact | Dock | Peptide Docking

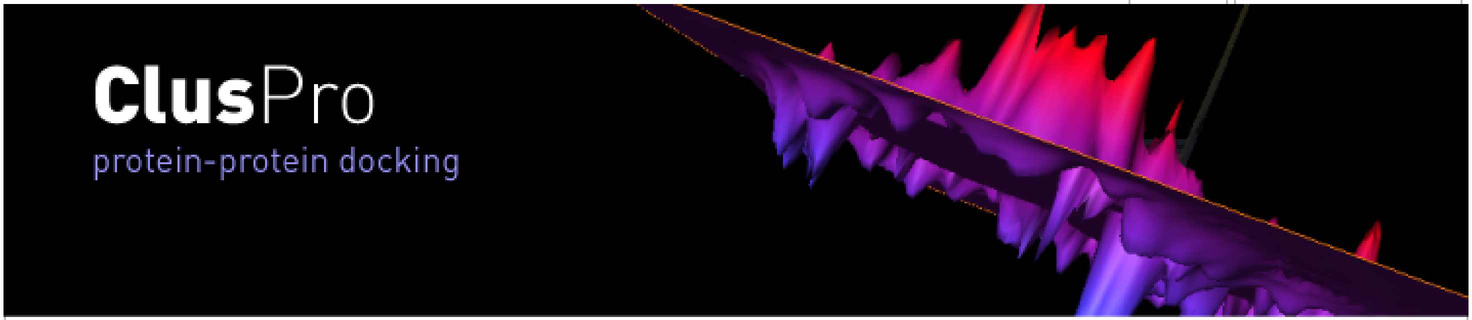

sign out

Job Details: 914958

View Models

Balanced | Electrostatic-favored | Hydrophobic-favored | VdW+Elec

Download Model Scores for this Coefficient

Coefficient Weights

See *Kozakov et. al.* in Papers for a description of these terms

$$E = 0.40E_{rep} + -0.40E_{att} + 600E_{elec} + 1.00E_{DARS}$$

Cluster Scores

We strongly encourage you to read the FAQ related to these scores before using them.

Cluster	Members	Representative	Weighted Score
**0**	42	Center	-1208.1
		Lowest Energy	-1588.8
**1**	37	Center	-1358.1
		Lowest Energy	-1586.6
**2**	25	Center	-1249.9
		Lowest Energy	-1411.4
**3**	25	Center	-1276.6
		Lowest Energy	-1708.9
**4**	24	Center	-1233.3
		Lowest Energy	-1656.2
**5**	23	Center	-1458.8
		Lowest Energy	-1666.1
**6**	23	Center	-1502.3
		Lowest Energy	-1564.3
**7**	21	Center	-1257.4

Cluster	Members	Representative	Weighted Score
		Lowest Energy	-1392.5
**8**	20	Center	-1469.8
		Lowest Energy	-1522.4
**9**	18	Center	-1167.8
		Lowest Energy	-1628.8
**10**	18	Center	-1219.1
		Lowest Energy	-1464.0
**11**	18	Center	-1254.5
		Lowest Energy	-1414.0
**12**	16	Center	-1226.8
		Lowest Energy	-1586.4
**13**	16	Center	-1255.0
		Lowest Energy	-1454.8
**14**	15	Center	-1168.0
		Lowest Energy	-1403.2
**15**	15	Center	-1265.7
		Lowest Energy	-1415.7
**16**	14	Center	-1154.9
		Lowest Energy	-1386.4
**17**	14	Center	-1325.8
		Lowest Energy	-1325.8
**18**	14	Center	-1310.1
		Lowest Energy	-1310.1
**19**	13	Center	-1239.2
		Lowest Energy	-1288.0
**20**	13	Center	-1341.8
		Lowest Energy	-1341.8
**21**	13	Center	-1329.6
		Lowest Energy	-1348.9
**22**	13	Center	-1277.3
		Lowest Energy	-1386.9
**23**	13	Center	-1227.0
		Lowest Energy	-1361.8
**24**	12	Center	-1188.7
		Lowest Energy	-1333.7
**25**	12	Center	-1220.5
		Lowest Energy	-1499.4

Cluster	Members	Representative	Weighted Score
**26**	12	Center	-1274.1
		Lowest Energy	-1578.7
**27**	12	Center	-1366.7
		Lowest Energy	-1366.7
**28**	12	Center	-1188.2
		Lowest Energy	-1239.7
**29**	11	Center	-1191.0
		Lowest Energy	-1571.6

ClusPro should only be used for noncommercial purposes.
Vajda Lab and ABC Group
Boston University and Stony Brook University
